# Interprofessional Coproduction of Diagnosis with Medical and Pharmacy Students: An Interactive Case-Based Workshop

**DOI:** 10.15766/mep_2374-8265.11437

**Published:** 2024-09-24

**Authors:** Valerie J. Lang, Melanie R. Symoniak, Sha-Phawn Williams

**Affiliations:** 1 Professor, Department of Medicine, University of Rochester School of Medicine and Dentistry; 2 Adjunct Professor, Wegmans School of Pharmacy, St. John Fisher University; 3 Clinical Assistant Professor of Pharmacy Practice, Howard University

**Keywords:** Case-Based Learning, Clinical Reasoning/Diagnostic Reasoning, Interprofessional Education, Pharmacy, Quality Improvement/Patient Safety

## Abstract

**Introduction:**

The Institute of Medicine and national competencies emphasize the importance of interprofessional education to reduce diagnostic error. Clinical pharmacists are increasingly integrated into clinical teams and participate in the diagnostic process. However, few educational resources explicitly teach medical and pharmacy students to collaborate on the production of diagnoses.

**Methods:**

We implemented a 2-hour, online, case-based workshop with 154 second-year medical students and third-year pharmacy students. After brief didactics on the diagnostic process and scope of practice of pharmacists, small groups of eight to 12 medical and pharmacy students with faculty facilitators worked through a case unfolding in two aliquots. Students were provided different but complementary information authentic to their profession. They had to communicate with each other to develop an appropriate differential diagnosis. Students then reflected on how communicating with the other profession impacted their diagnostic reasoning. Comments were coded and counted.

**Results:**

The majority (99%) of students identified their data gathering and differential diagnoses were impacted by working through the case together. More pharmacy students commented on how medical students broadened their differential diagnosis (71%) and added information (72%), contextualizing information, such as past history, medication indications, and physical exam data. More medical students commented on how pharmacy students helped justify (54%) and clarify (22%) the differential diagnosis, often connecting the underlying mechanism of medications with clinical findings.

**Discussion:**

This interactive case-based workshop was effective in teaching medical and pharmacy students to collaborate in the coproduction of diagnosis. It is feasible with minimal resources.

## Educational Objectives

By the end of this activity, learners will be able to:

1. Identify the roles and responsibilities of pharmacists and physicians in the diagnostic reasoning process.
2. Identify the scope of practice for pharmacists.
3. Communicate with members of an interprofessional team to coproduce a diagnosis.

## Introduction

Diagnostic error is common, occurring in approximately 15% of outpatient primary care visits.^1^ When patients present with an undifferentiated symptom, the differential diagnosis often includes adverse events related to their medications, especially in the setting of polypharmacy. Adverse events, with or without medication errors, create common diagnostic challenges for interprofessional teams caring for older patients. The transition from hospitalization to outpatient care is a particularly high-risk period, with approximately 50% of adults experiencing medication errors and 20% experiencing adverse drug events^2^ requiring diagnosis. Diagnosis is a multifaceted process that includes: (1) data gathering (e.g., history and physical exam) and interpretation; (2) synthesis of a case into a problem representation; (3) generation, prioritization, and justification of a differential diagnosis (linking the findings from the history, physical exam, tests, and knowledge of underlying physiology to generate hypotheses about the diagnosis); and (4) selection of a leading/working diagnosis and diagnostic plan.^3^ While physicians have long been recognized for their role in diagnosis, pharmacists are increasingly integrated into clinical teams.^4,5^ Pharmacists are considered one of the most accessible health care professionals, with nearly 90% of the population living within five miles of a pharmacy,^6^ and often times patients are able to seek health care advisement from a pharmacist before being able to be seen by their primary care provider. While most pharmacists are not explicitly taught diagnostic reasoning, they participate in many facets of the diagnostic process,^7^ including history data gathering (e.g., when patients discuss side effects of medications with their pharmacist), laboratory data interpretation (e.g., as part of monitoring for medication toxicity), and differential diagnosis generation and justification (e.g., for symptoms that may be caused by medication side effects or nonadherence, developing a list of diagnostic hypotheses and linking data about the case with knowledge about medications’ underlying mechanisms of action and pharmacokinetics).

There is increasing interest in more explicitly training pharmacy students about their role in the diagnostic process^7,8^ and training pharmacists and physicians to collaborate in the diagnostic process^7–9^ (coproduction of diagnosis). In 2015, the Institute of Medicine issued the *Improving Diagnosis in Healthcare* report^10^ in which the eight goals to reduce diagnostic error included: (1) “facilitate more effective teamwork in the diagnostic process among health care professionals, patients, and their families”; and (2) “enhance health care professional education and training in the diagnostic process.”^10^ Similarly, both the AAMC's 13 Core Entrustable Professional Activities for Entering Residency and the American Association of Colleges of Pharmacy's Core Entrustable Professional Activities for New Pharmacy Graduates include “collaborate as a member of an interprofessional team.”^11,12^ The Interprofessional Education Collaborative competencies also include “use the knowledge of one's own role and those of other professions to appropriately assess and address the health care needs of patients and to promote and advance the health of populations.”^13^ Lastly, the Society to Improve Diagnosis in Medicine convened education leaders in a variety of health professions, including medicine and pharmacy, and developed interprofessional competencies more specifically targeted to diagnosis, including “collaborate with other health care professionals (including nurses, physicians, physician assistants, radiologists, laboratory professionals, pharmacists, social workers, physical therapists, medical librarians, and others) and communicate effectively throughout the diagnostic process.”^9^

To achieve these interprofessional competencies in diagnosis, well-designed and feasible educational activities are needed. Several interprofessional education activities that include medical students and pharmacy students in some facet of diagnosis have been published. Activities include high-fidelity simulations using mannequins,^14^ standardized patients,^15^ or both.^16^ However, high-fidelity simulations are resource intensive and not always necessary to meet learning objectives focused on diagnostic data sharing, interpretation, and differential diagnosis. In another published innovation, the authors used a low-fidelity case discussion focused on patient safety for medical, nursing, and pharmacy students that involved development of a differential diagnosis; the exercise increased students’ awareness of communication errors and social or cultural factors that contribute to patient care error.^17^ Still another innovation describes interprofessional teams of medical, social work, nursing, and pharmacy students working through a case asynchronously, using and recording information in a mock electronic health record.^18^ However, the cost of the software (approximately $200,000)^18^ poses a potential constraint on implementation at other institutions. There is a need for more experiential educational activities designed specifically to promote communication between medical and pharmacy students in coproducing a diagnosis and that can be delivered with standard resources.

The overarching goal of this interactive workshop was to engage medical students and pharmacy students in a case that required them to communicate with each other, sharing information that was specific to their professions, in order for them to learn experientially how to coproduce a differential diagnosis. The workshop was designed around a common patient scenario related to medication prescribing that was appropriate for medical students and pharmacy students.

## Methods

We delivered the 2-hour, interprofessional workshop online by videoconference with medical students and pharmacy students (Appendix A). Medical students were at the end of their second year; they had completed a 1-year primary care clerkship during which they participated in didactic sessions on primary care management of common acute and chronic diagnoses and saw patients in primary care offices for 38 half-days. Medical students had also completed the majority of a disease process and therapeutics course and were preparing to start inpatient clerkships. While medical students were likely to identify as diagnosticians, their interactions with clinical pharmacists were likely highly variable, depending on the office setting in which they were placed. They had previously participated in three other interprofessional education sessions, including two with social work students and one with nursing students; however, this was the first interaction between the pharmacy students and medical students.

Pharmacy students were nearing the end of their third year; they were completing the last semester of a four-semester pathophysiology and therapeutics course sequence and were preparing to enter the advanced experiential year. Throughout their curriculum, pharmacy students were not taught to diagnose, but to understand the epidemiology, etiology, pathophysiology, clinical signs, symptoms, diagnosis measures, and relevant biomarkers related to a disease to fully understand why a specific medication is recommended. Students then learned how to implement therapy by applying guideline-driven treatment options from the most updated and widely used guidelines. Pharmacy students were also taught how to assess if a patient is a candidate for self-care treatment or if a patient is experiencing a medical emergency. Pharmacy students participate in nine required interprofessional education sessions over the 4-year curriculum, including two sessions with physician assistant students, four sessions with nursing students, two sessions with medical students, one session with physical therapy and occupational therapy students, and a Team Strategies and Tools to Enhance Performance and Patient Safety (TeamSTEPPS)^19^ training with nursing and graduate mental health counseling students.

### Large-Group Session (0-30 Minutes)

We provided a 15-minute didactic lecture on the diagnostic process and the incidence and impact of diagnostic error (Appendix B) followed by a 15-minute didactic lecture on the scope of practice of clinical pharmacists (Appendix C).

### Small-Group Case Exercise (30-110 Minutes)

#### Facilitators

Each small group of eight to 12 students was facilitated by a pharmacy or medical faculty member. We provided facilitators with a detailed facilitator guide (Appendix D) and held a 30-minute orientation to discuss the exercises and address questions. During the orientation, we discussed the goals of the workshop and reviewed the facilitator guide and small-group exercise step-by-step. We emphasized the importance of an ice breaker to develop psychological safety among the small groups, identified the points at which facilitators should guide the students to read each aliquot and submit their individual reflections, and highlighted strategies for encouraging students to participate if conversations stalled.

#### Interactive case

Faculty members from medicine and pharmacy collaboratively developed a case of an older adult on multiple medications who developed new symptoms after discharge from the hospital. The case unfolded in two aliquots as new information was obtained, and we provided students with different information according to their profession (Appendices E–H). The pharmacy students were given information that would typically be available to a community pharmacist, including names, doses, quantities, and dates of outpatient medication fills and refills, and history information provided by the patient's caregiver who approaches the pharmacist with questions. The medical students were given information that would typically be available to a primary care physician, including past medical history, a prehospitalization medication list, hospital discharge summary, and a telephone call from the visiting nurse.

After the first large-group session, we divided the students up into breakout rooms with groups of eight to 12, each with medical students and pharmacy students and a faculty facilitator from either the medical school or pharmacy school. After the first of the two case aliquots, students completed an individual exercise (Appendix I) and then shared their thoughts during their small-group discussion. At the end of the case, students completed an individual reflection describing how communicating with their interprofessional counterparts impacted their diagnostic reasoning, if at all (Appendix J).

We used a learning management system and a cloud-based system to distribute the case aliquots and for students to submit their assignments.

### Large-Group Wrap-up (110-120 Minutes)

We gathered the students back from their breakout rooms for a 10-minute wrap-up, including an expert clinician explanation of their diagnostic reasoning of the case (Appendix K).

### Evaluation

We analyzed students’ reflections on how communicating with students of the other profession impacted their diagnostic process, if at all. The codes were developed inductively. Two authors (Valerie Lang and Melanie Symoniak) independently reviewed the reflections and developed codes and subsequently met to finalize a codebook and definitions. Next, both investigators independently applied the final codes to the students’ responses. They met and resolved any discrepancies through discussion.

We debriefed facilitators immediately after the intervention and incorporated their suggestions into the next year's facilitator guide.

### Ethical Approval

The project was submitted to the institutional review boards at both institutions (St. John Fisher University Institutional Review Board [File No. 4216-012022-01] and University of Rochester Research Subjects Review Board Exemption [STUDY00006825]) and determined to be exempt from full review.

## Results

Fifty-four pharmacy students and 100 medical students participated in the workshop. Of these, 51 (94%) pharmacy students and 99 (99%) medical students submitted their reflections. The majority of students commented on how their data gathering and differential diagnoses were impacted by working through the case together (Table 1). More pharmacy students commented on how medical students broadened their differential diagnosis and added information than vice versa, and more medical students commented on how pharmacy students helped justify and clarify (reprioritize) their differential diagnosis than vice versa. Representative comments are included in Table 2. In their reflections, students also described how participating in the exercise shifted their appreciation for each other's roles. Representative comments are included in Table 3.

**Table 1.**
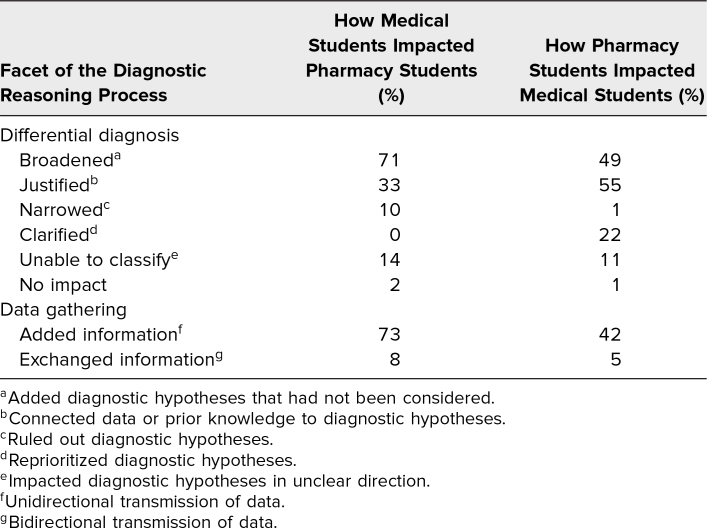
Pharmacy Students’ (*N* = 51) and Medical Students’ (*N* = 99) Descriptions of How Communicating With the Other Profession Impacted Their Own Diagnostic Process

**Table 2.**
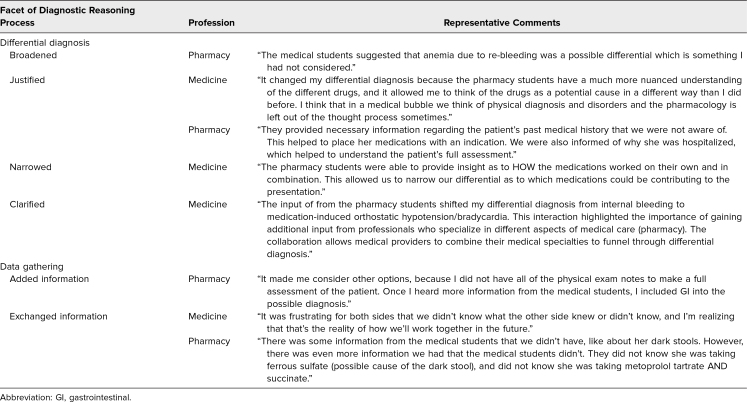
Representative Comments Regarding Facets of the Diagnostic Reasoning Process Impacted by Communicating With Students in the Other Profession

**Table 3.**
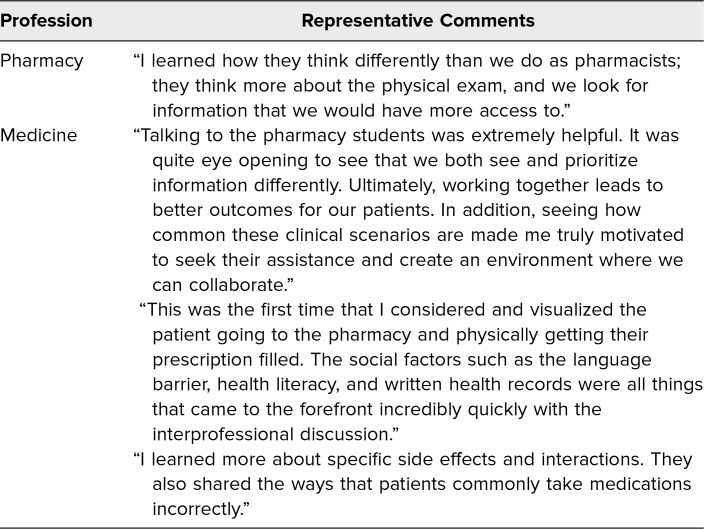
Representative Comments From Students’ Reflections About Their Appreciation for Each Other's Roles

## Discussion

Our interactive case-based workshop successfully engaged pharmacy and medical students in the process of coproduction of diagnosis. Students identified how working with students from the other profession impacted their diagnostic process, but in different ways. Pharmacy students were more likely to broaden their differential diagnoses beyond medication-related phenomena based on discussion with medical students, and medical students were more likely to justify and clarify their differential diagnoses, typically by improving the connection between medication actions and clinical findings, based on discussion with pharmacy students.

This workshop could be implemented at other institutions in the appropriate context. Compared to high-fidelity simulations, this exercise was feasible with minimal resources. The primary resource was small-group facilitators, with one faculty member per eight to 12 students spending approximately 2 hours for preparation and facilitation. We implemented and conducted the workshop by video conference, which required administrative support to manage the breakout rooms, but it could also be conducted in a face-to-face setting.

The success of this workshop was due in part to the collaboration between faculty leaders at the medical school and the pharmacy school, timing the case-based workshop to an appropriate point in each profession's curriculum, and incorporating the workshop into existing courses within each program. While the pharmacists’ information could have been simply distributed to the medical students, the experience of communicating with the other profession fostered opportunities to learn from each other and simulated the types of conversations that physicians and pharmacists have in authentic clinical practice. For students to contribute meaningfully to the discussion, the pharmacy students needed sufficient knowledge of drug actions, interactions and side effects as well as treatment guidelines, and how to interpret a dispensing schedule, and medical students needed sufficient knowledge of disease pathology, basic pharmacology, normal vital signs, and how to interpret outpatient medical records.

Facilitator faculty development is also important to the success of interprofessional education.^20,21^ By combining a detailed facilitator guide with an interactive orientation session, faculty members from each profession were able to successfully facilitate the small-group discussions.

There were some limitations to implementing the activity. Although medical students had already been introduced to how to construct a summary statement, this was a novel exercise for pharmacy students. Medical students outnumbered pharmacy students, and they had not previously worked together, which could have affected student comfort and engagement in the small-group discussion. Although both medical and pharmacy students recognized the impact of the interprofessional experience on their diagnostic reasoning, it is possible that there was a greater impact on the medical students, who had fewer formal interprofessional experiences than the pharmacy students prior to this workshop. Finally, although students reflected on what they learned about each other's roles, we did not quantitively assess knowledge about scope of practice. We proceeded with the assumption that most pharmacy students are familiar with the physician scope of practice, but medical students may not be as familiar with the expanding pharmacist scope of practice and abilities. Future evaluations could explore this assumption further.

Because of the success of the interprofessional workshop, the medical school and pharmacy school are continuing the workshop with future cohorts and collaborating on plans for additional interprofessional education activities in other areas of the curriculum.

## Appendices


Session Outline for Students.docxIntro to Diagnostic Error and IP Dx.pptxPharmacist Scope of Practice.pptxInterprofessional Case Facilitator Guide.docxAliquot 1 for Medical Students.docxAliquot 1 for Pharmacy Students.docxAliquot 2 for Medical Students.docxAliquot 2 for Pharmacy Students.docxIndividual Reflection After Aliquot 1.docxIndividual Reflection After Aliquot 2.docxWrap-up Session Slides.pptx
*All appendices are peer reviewed as integral parts of the Original Publication.*
