# Supplementary material for: Interprofessional Coproduction of Diagnosis with Medical and Pharmacy Students: An Interactive Case-Based Workshop
Source: MedEdPORTAL. 2024 Sep 24;20:11437. doi: 10.15766/mep_2374-8265.11437 (PMC11402627; doi:10.15766/mep_2374-8265.11437)
Supplement: Supplementary file 1 — Session Outline for Students.docxIntro to Diagnostic Error and IP Dx.pptxPharmacist Scope of Practice.pptxInterprofessional Case Facilitator Guide.docxAliquot 1 for Medical Students.docxAliquot 1 for Pharmacy Students.docxAliquot 2 for Medical Students.docxAliquot 2 for Pharmacy Students.docxIndividual Reflection After Aliquot 1.docxIndividual Reflection After Aliquot 2.docxWrap-up Session Slides.pptx [file mep_2374-8265.11437-s001.zip › A. Session Outline for Students.docx]

**Interprofessional Co-production of Diagnosis**

**Session Outline for Medical and Pharmacy Students**

**Introduction**

In this 2-hour session, medical students and medical school faculty will join with pharmacy students and faculty. You’ll learn about the scope of practice of clinical pharmacists and then work collaboratively with senior pharmacy students on a diagnostic challenge. You’ll experience how physicians and pharmacists work together as members of interprofessional teams to contribute to patient safety.

**Prework:** None

| **Learning objectives for this session (All)** | **IPEC competency (Pharmacy Students)** | **Medical Student Course Goals** |
| --- | --- | --- |
| Identify the roles and responsibilities of pharmacists and physicians in the diagnostic reasoning process | Engage diverse professionals who complement one's own professional expertise, as well as associated resources, to develop strategies to meet specific health and healthcare needs of patients and populations.  RR9. Use unique and complementary abilities of all members of the team to optimize health and patient care. | Appreciate the importance of inter-professional teamwork in providing patient-centered care.  Integrate the contributions of patients, caregivers, and other IP team members to co-produce diagnoses effectively. |
| Identify the scope of practice for pharmacists under CDTM | Explain the roles and responsibilities of other providers and how the team works together to provide care, promote health, and prevent disease. | Appreciate the importance of inter-professional teamwork in providing patient-centered care. |
| Communicate with members of an interprofessional team to co-produce a diagnosis | Express one's knowledge and opinions to team members involved in patient care and population health improvement with confidence, clarity, and respect, working to ensure common understanding of information, treatment, care decisions, and population health programs and policies.  Communicate the importance of teamwork in patient-centered care and population health programs and policies. | Work with individuals of other professions to maintain a climate of mutual respect.  Listen actively, and encourage ideas from other team members.  Appreciate the importance of inter-professional teamwork in providing patient-centered care.  Integrate the contributions of patients, caregivers, and other IP team members to co-produce diagnoses effectively. |

**15 minutes Large Group:**

Introduction to diagnosis and diagnostic error

Introduction to the session

**15 minutes Large Group:** Roles and responsibilities: Scope of practice of pharmacists

**80 minutes Small Group*:***

**Case exercise**: simulation of interprofessional collaborative practice

**10 minutes: Large group:** Reveal- Expert discussion of case

**Optional Reading**

1. Graber ML, Grice GR, Ling LJ, Conway JM, Olson A. Pharmacy education needs to address diagnostic safety. *Am J Pharm Educ*. 2019;83(6):1179-1188. <https://doi.org/10.5688/ajpe7442>
2. Nelson NR, Jones M, Wilbur LG, Romanelli F. Making diagnostic instruction explicit in US pharmacy education. *Am J Pharm Educ*. 2020;84(5):7791. <https://doi.org/10.5688/ajpe7791>
3. Cahill M, Gleason K, Harkless G, Stanley J, Graber M. The regulatory implications of engaging registered nurses in diagnoses. *J Nurs Regulation*. 2019;10(2):5-10. <https://doi.org/10.1016/S2155-8256(19)30110-3>
4. Balogh EP, Miller BT, Ball JR, eds; Committee on Diagnostic Error in Health Care, Board on Health Care Services, Institute of Medicine, National Academies of Sciences, Engineering, and Medicine. *Improving Diagnosis in Health Care*. National Academies Press; 2015. Accessed June 21, 2024. <https://nap.nationalacademies.org/catalog/21794/improving-diagnosis-in-health-care>
