# Supplementary material for: Interprofessional Coproduction of Diagnosis with Medical and Pharmacy Students: An Interactive Case-Based Workshop
Source: MedEdPORTAL. 2024 Sep 24;20:11437. doi: 10.15766/mep_2374-8265.11437 (PMC11402627; doi:10.15766/mep_2374-8265.11437)
Supplement: Supplementary file 1 — Session Outline for Students.docxIntro to Diagnostic Error and IP Dx.pptxPharmacist Scope of Practice.pptxInterprofessional Case Facilitator Guide.docxAliquot 1 for Medical Students.docxAliquot 1 for Pharmacy Students.docxAliquot 2 for Medical Students.docxAliquot 2 for Pharmacy Students.docxIndividual Reflection After Aliquot 1.docxIndividual Reflection After Aliquot 2.docxWrap-up Session Slides.pptx [file mep_2374-8265.11437-s001.zip › B. Intro to Diagnostic Error and IP Dx.pptx]

## Slide 1
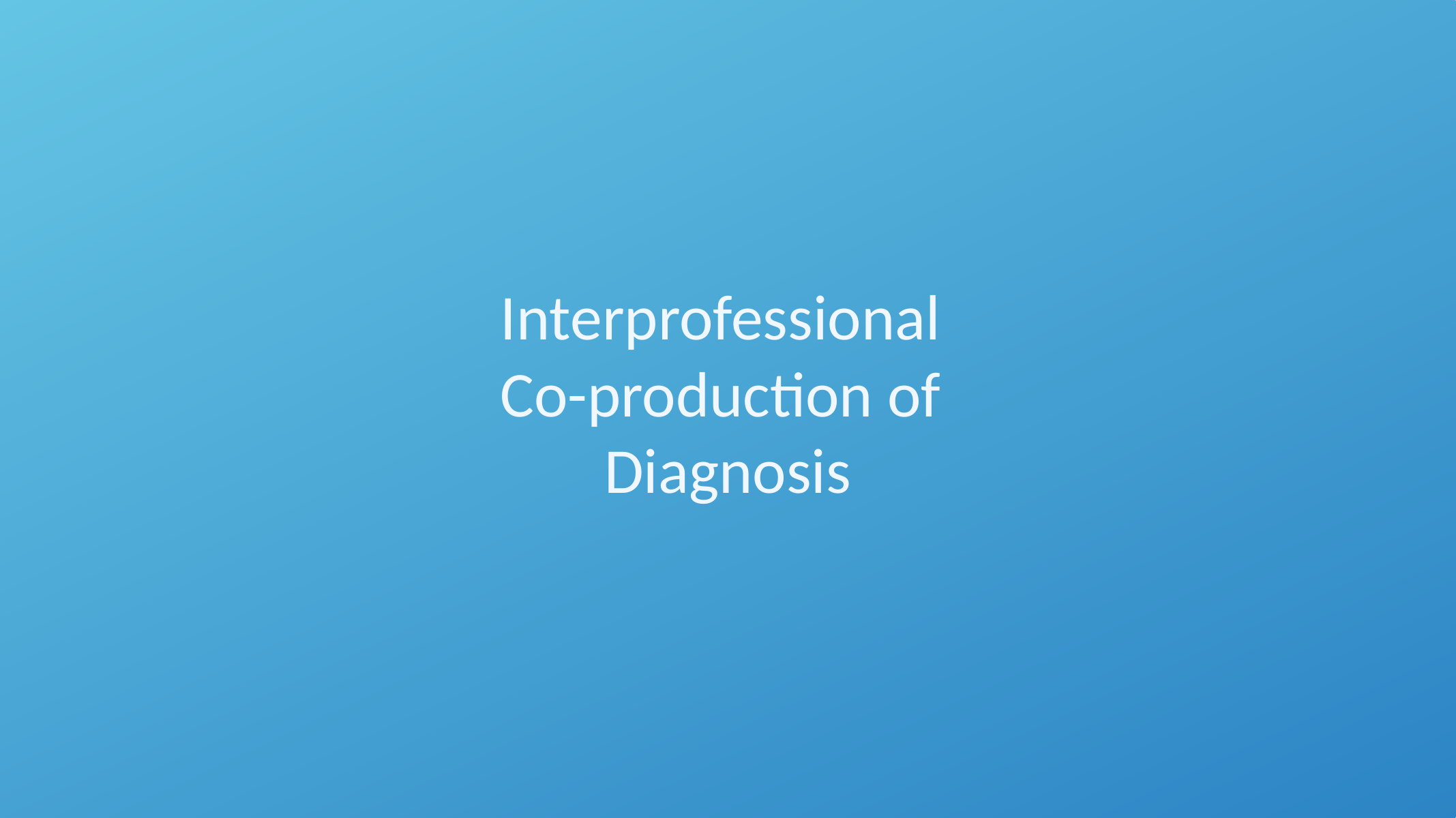

Interprofessional
Co-production of
Diagnosis

## Slide 2
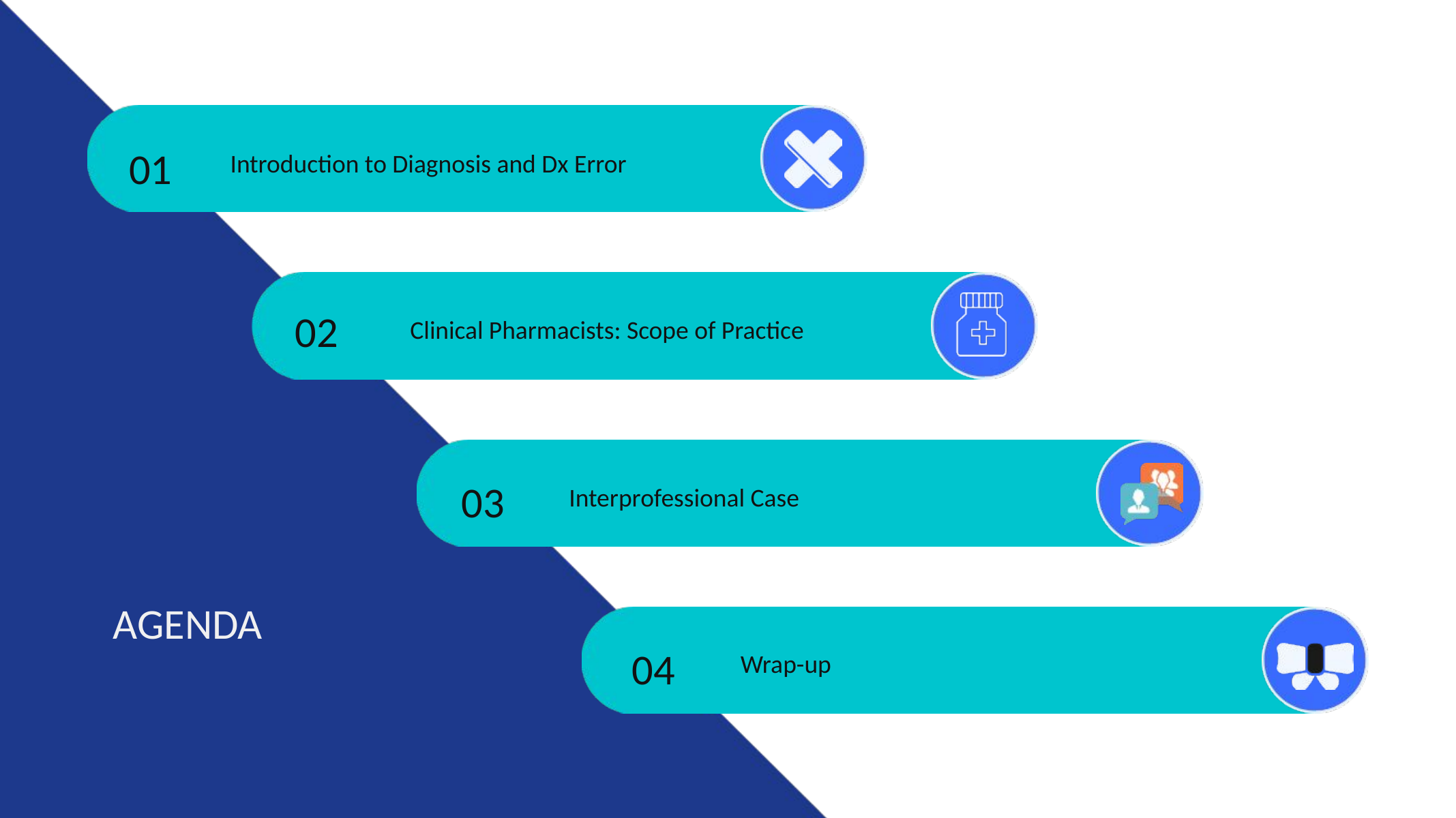

01
Introduction to Diagnosis and Dx Error
02
Clinical Pharmacists: Scope of Practice
03
Interprofessional Case
AGENDA
04
Wrap-up

## Slide 3
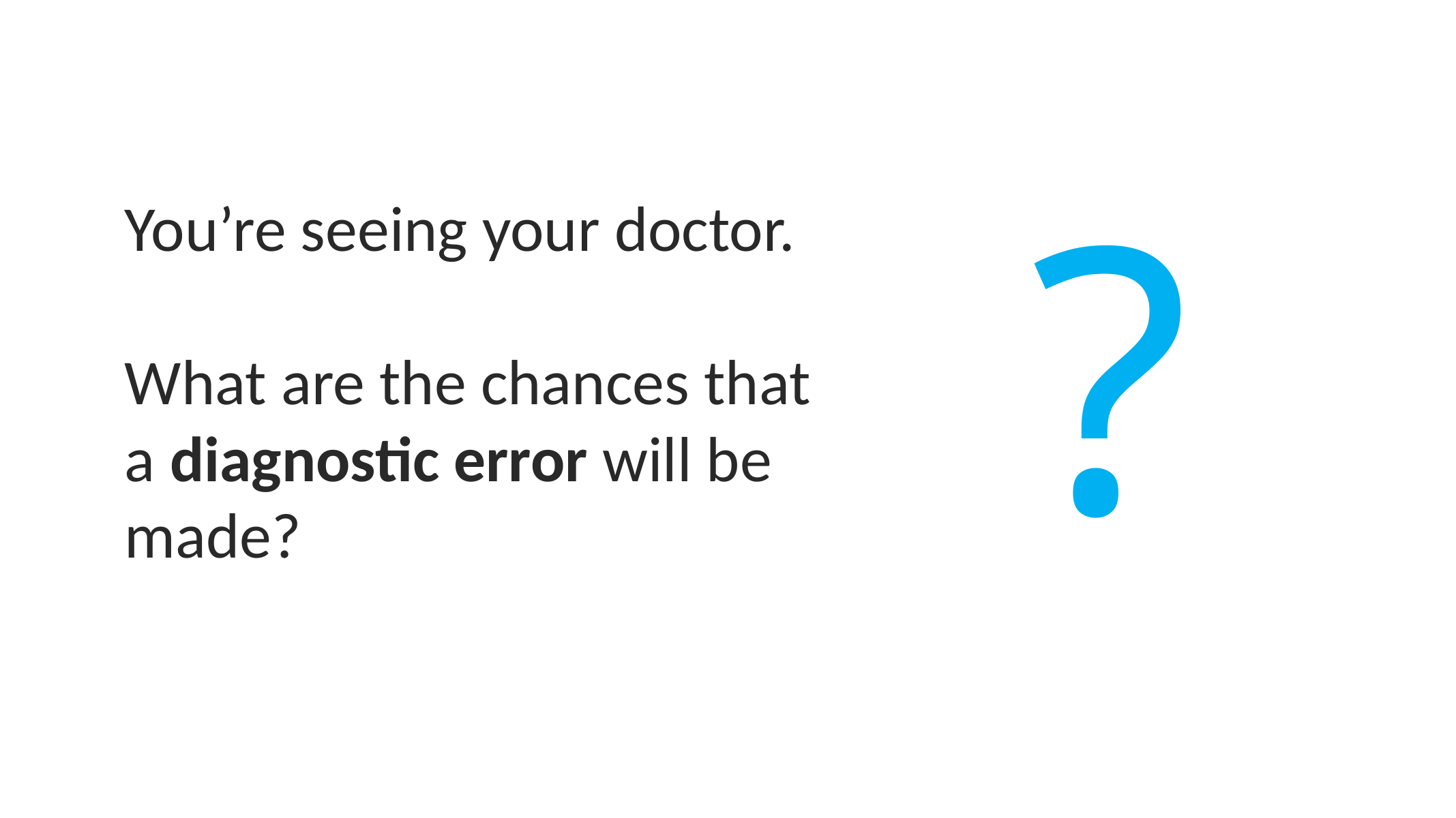

?
You’re seeing your doctor.
What are the chances that a diagnostic error will be made?

## Slide 4
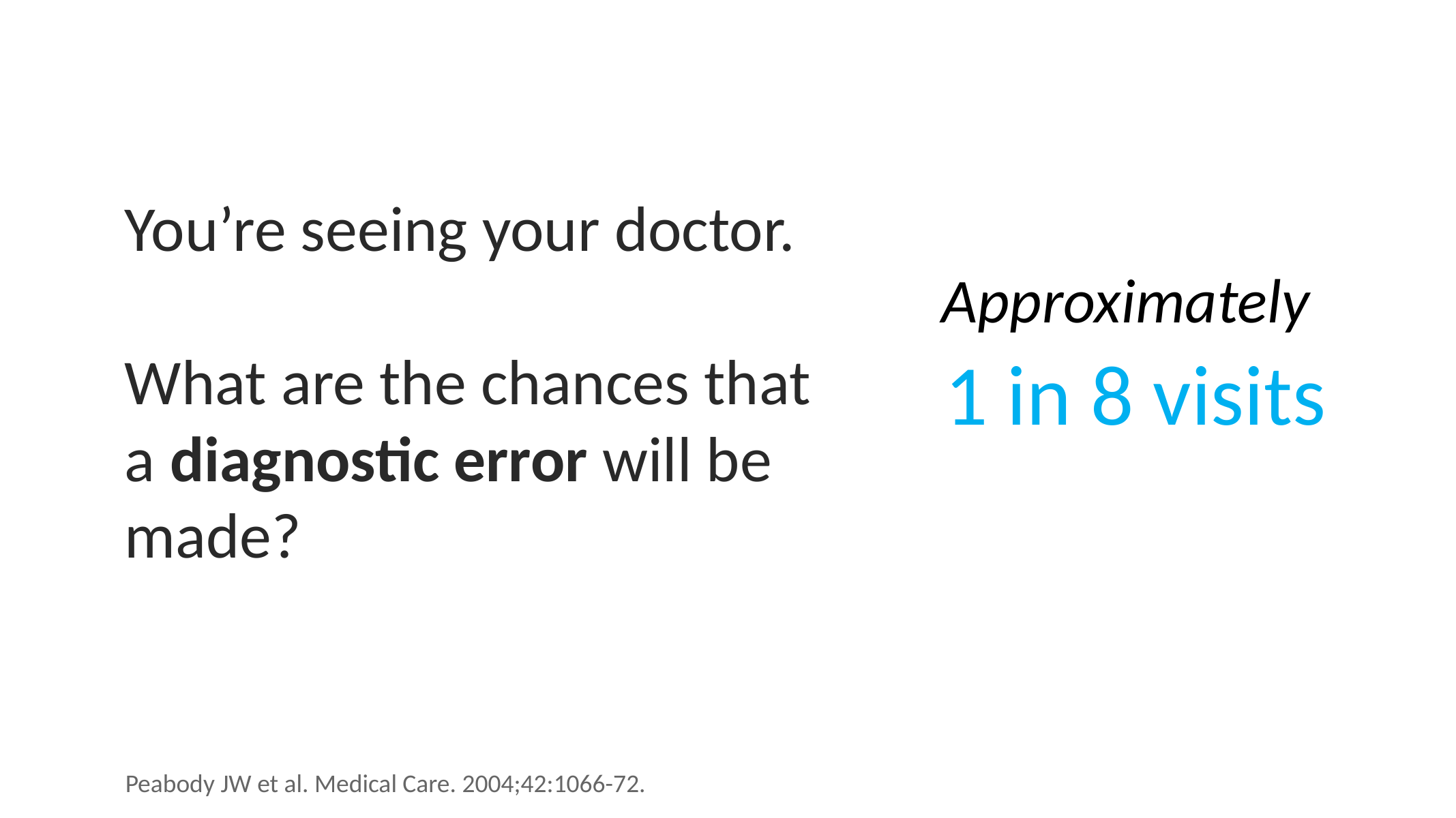

You’re seeing your doctor.
What are the chances that a diagnostic error will be made?
Approximately
1 in 8 visits
Peabody JW et al. Medical Care. 2004;42:1066-72.

## Slide 5
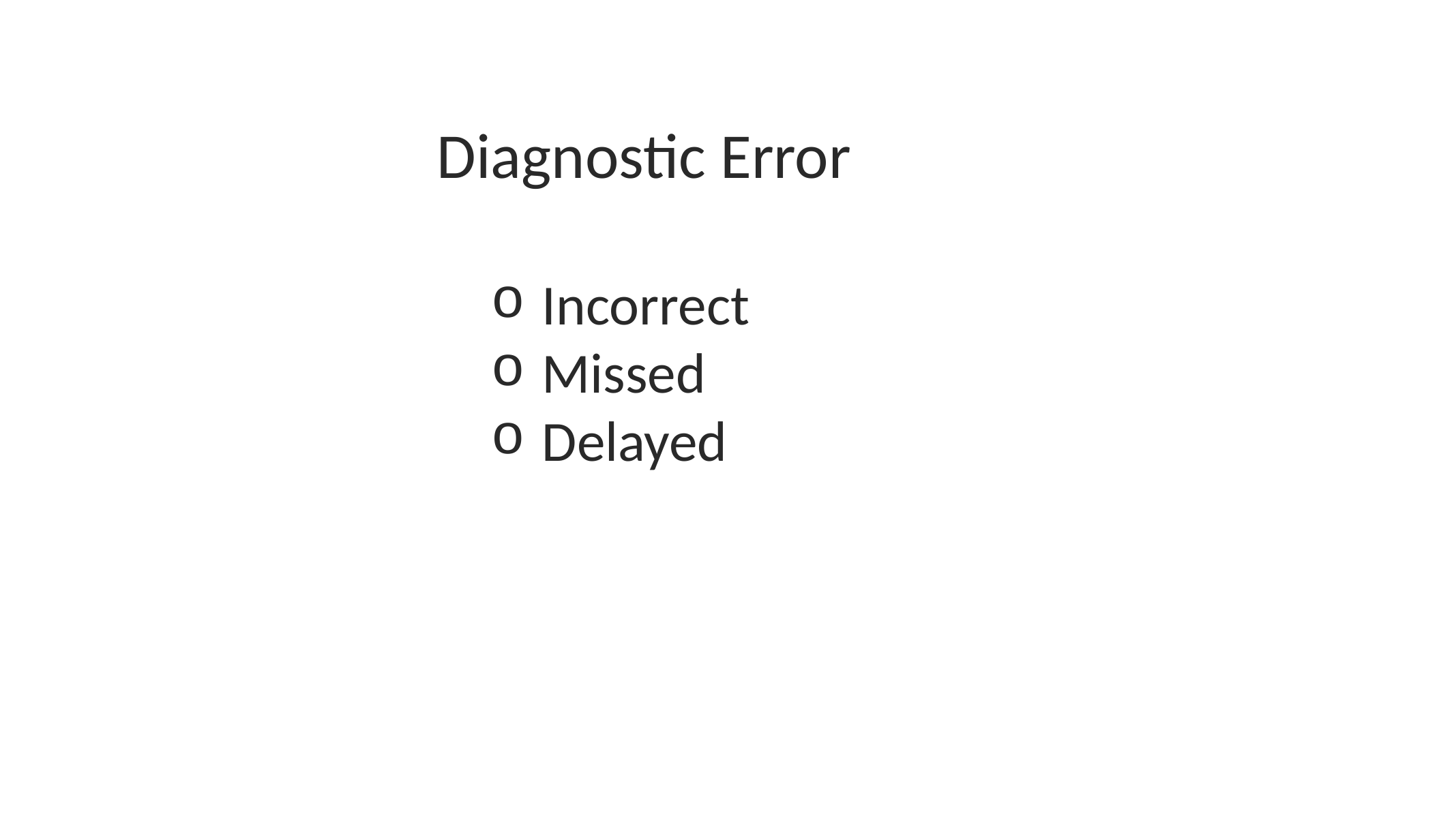

Diagnostic Error
Incorrect
Missed
Delayed

## Slide 6
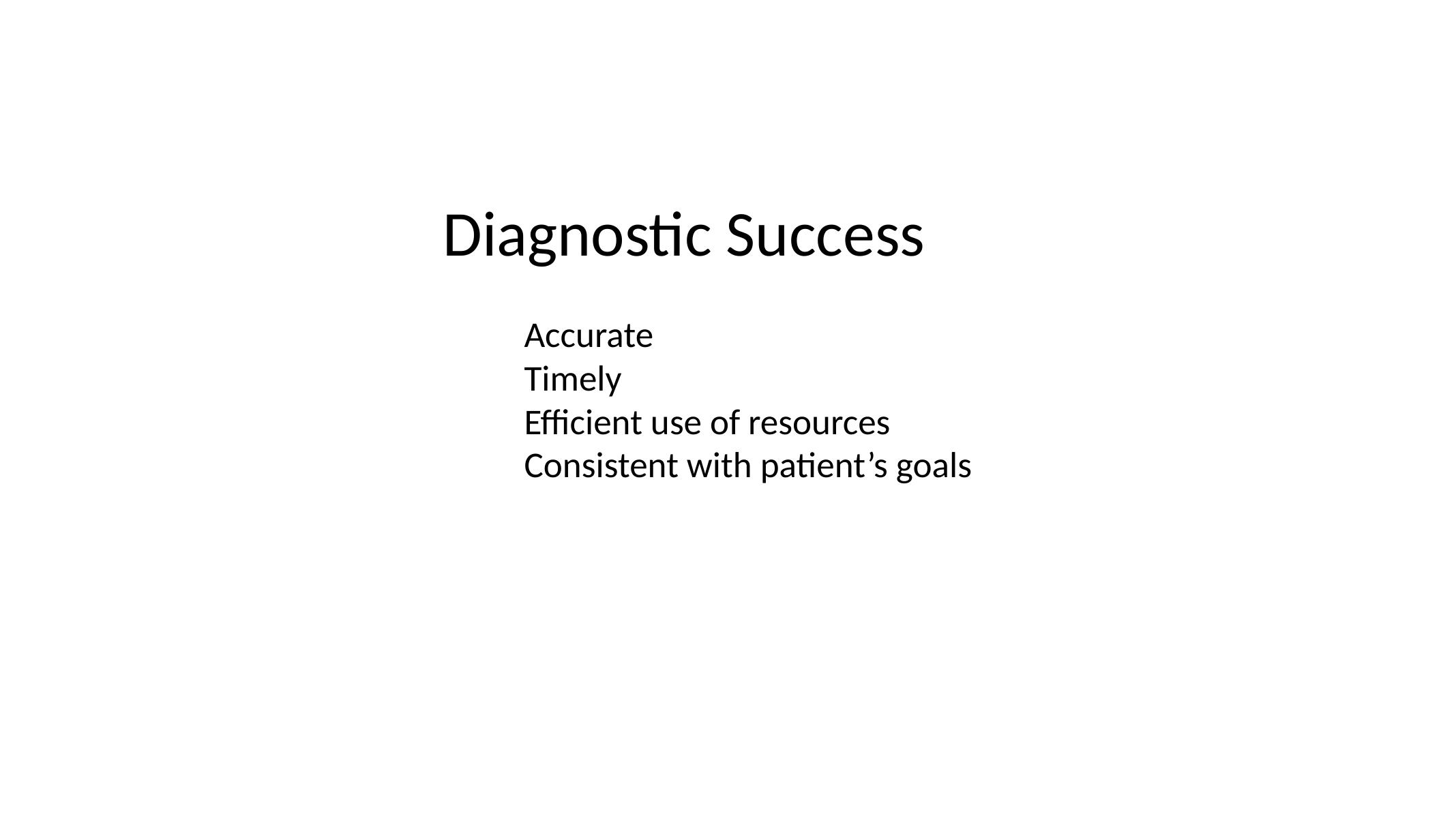

Diagnostic Success
Accurate
Timely
Efficient use of resources
Consistent with patient’s goals

## Slide 7
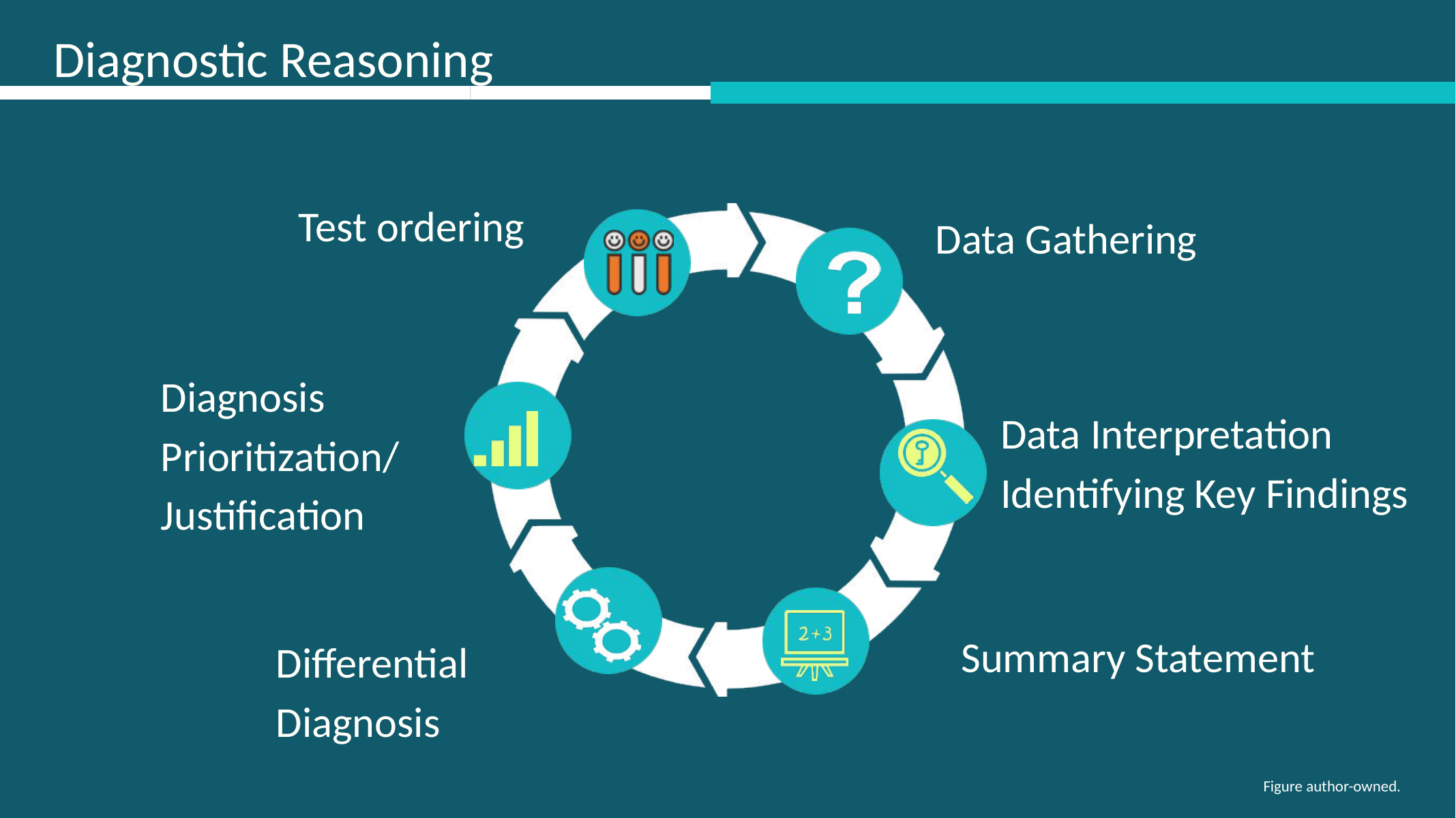

Diagnostic Reasoning
Test ordering
Data Gathering
Diagnosis Prioritization/
Justification
Data Interpretation
Identifying Key Findings
Summary Statement
Differential Diagnosis
Figure author-owned.

## Slide 8
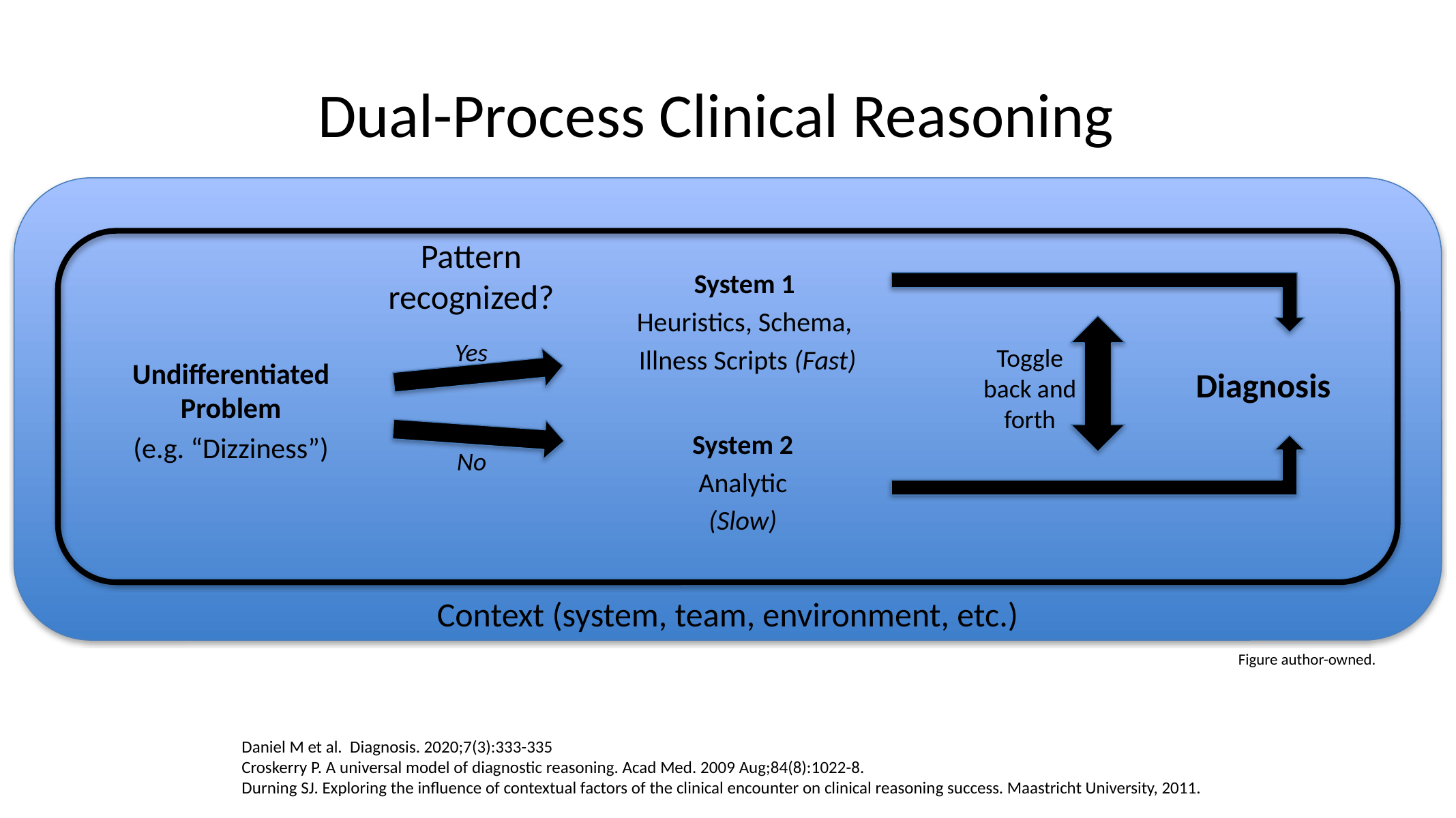

# Dual-Process Clinical Reasoning
Pattern recognized?
System 1
Heuristics, Schema,
 Illness Scripts (Fast)
Yes
Toggle back and forth
Undifferentiated Problem
(e.g. “Dizziness”)
Diagnosis
System 2
Analytic
(Slow)
No
Context (system, team, environment, etc.)
Figure author-owned.
Daniel M et al. Diagnosis. 2020;7(3):333-335
Croskerry P. A universal model of diagnostic reasoning. Acad Med. 2009 Aug;84(8):1022-8.
Durning SJ. Exploring the influence of contextual factors of the clinical encounter on clinical reasoning success. Maastricht University, 2011.

## Slide 9
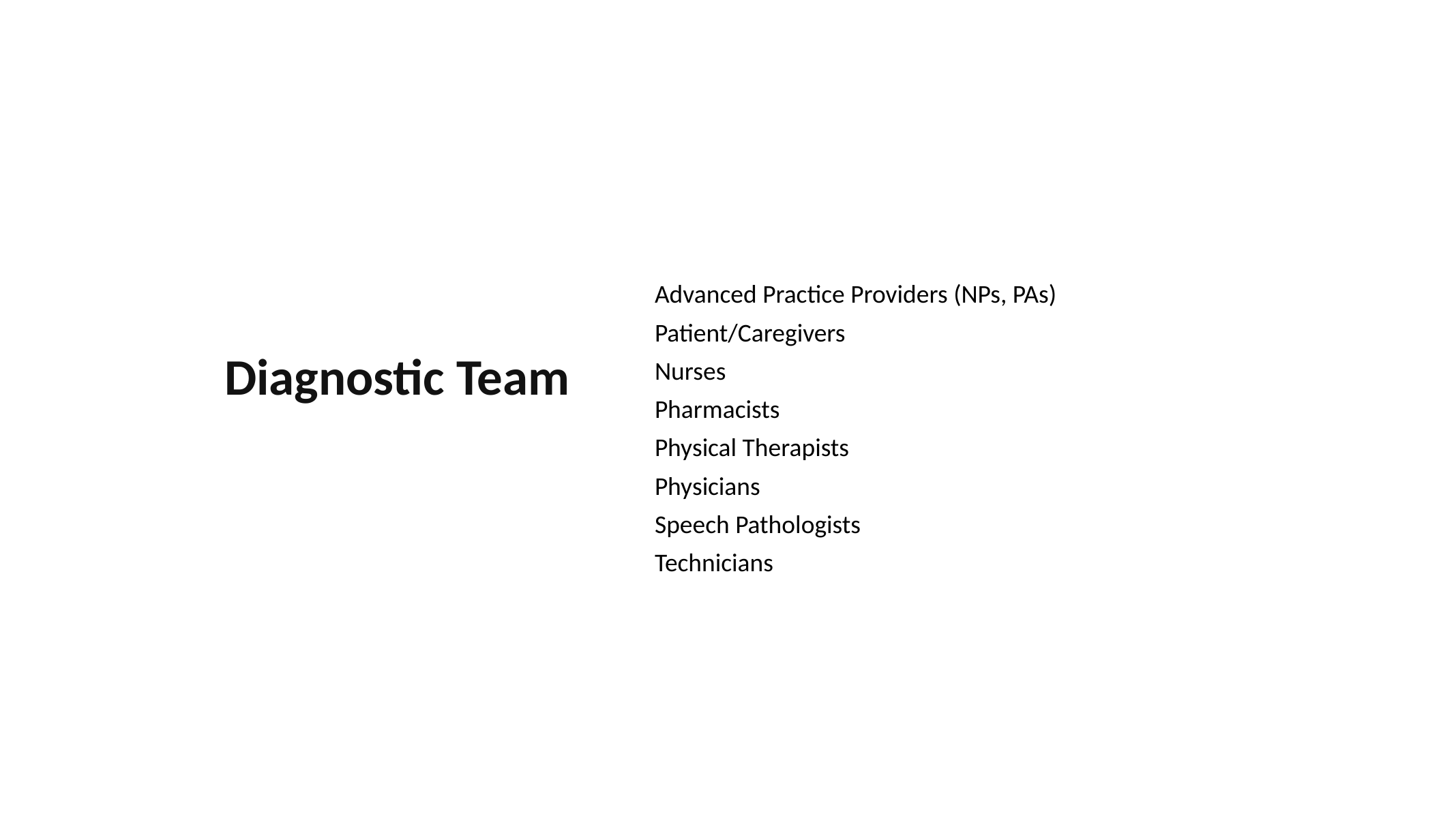

Advanced Practice Providers (NPs, PAs)
Patient/Caregivers
Nurses
Pharmacists
Physical Therapists
Physicians
Speech Pathologists
Technicians
Diagnostic Team

## Slide 10
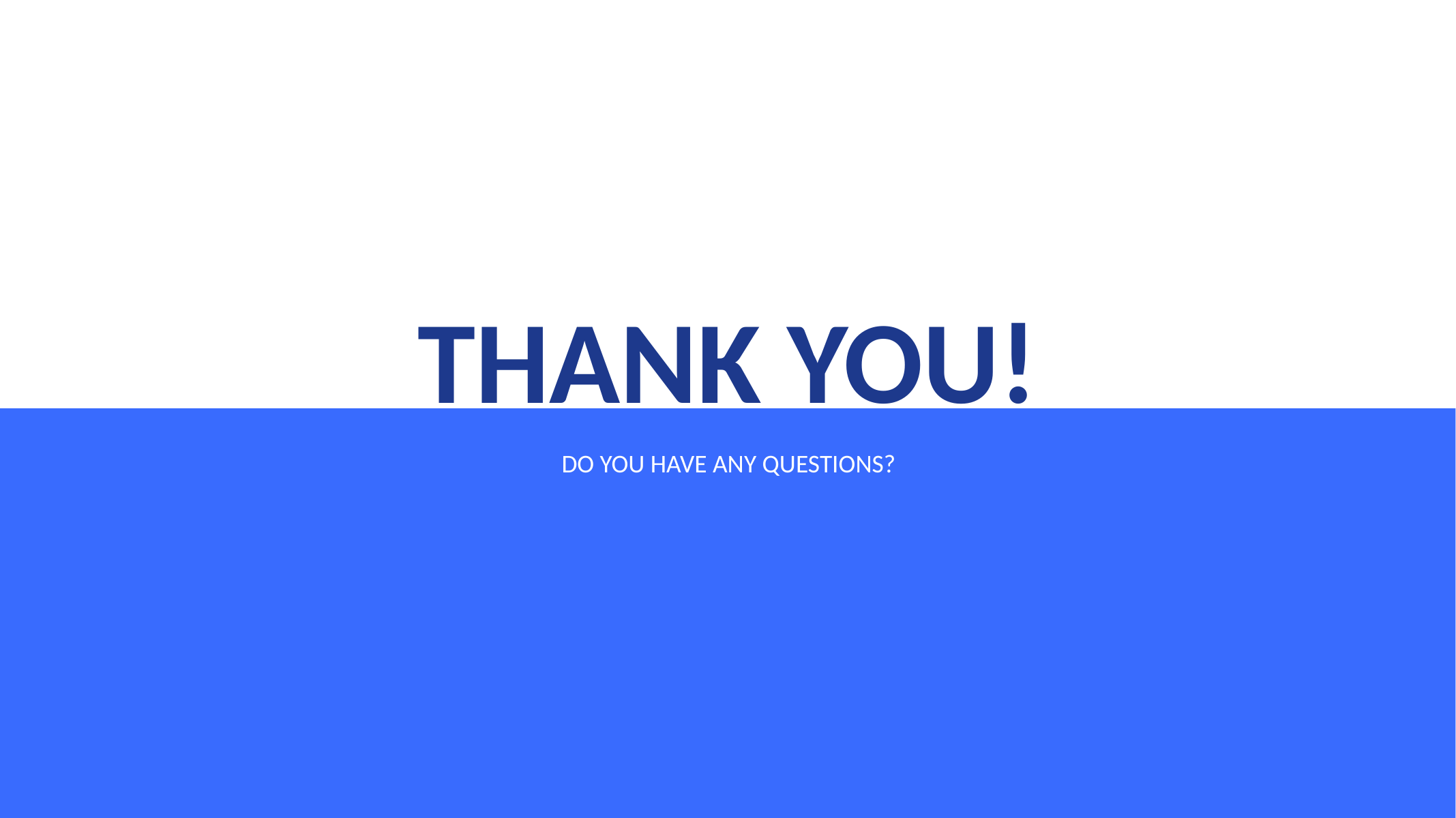

THANK YOU!
DO YOU HAVE ANY QUESTIONS?
