# Supplementary material for: Interprofessional Coproduction of Diagnosis with Medical and Pharmacy Students: An Interactive Case-Based Workshop
Source: MedEdPORTAL. 2024 Sep 24;20:11437. doi: 10.15766/mep_2374-8265.11437 (PMC11402627; doi:10.15766/mep_2374-8265.11437)
Supplement: Supplementary file 1 — Session Outline for Students.docxIntro to Diagnostic Error and IP Dx.pptxPharmacist Scope of Practice.pptxInterprofessional Case Facilitator Guide.docxAliquot 1 for Medical Students.docxAliquot 1 for Pharmacy Students.docxAliquot 2 for Medical Students.docxAliquot 2 for Pharmacy Students.docxIndividual Reflection After Aliquot 1.docxIndividual Reflection After Aliquot 2.docxWrap-up Session Slides.pptx [file mep_2374-8265.11437-s001.zip › C. Pharmacist Scope of Practice.pptx]

## Slide 1
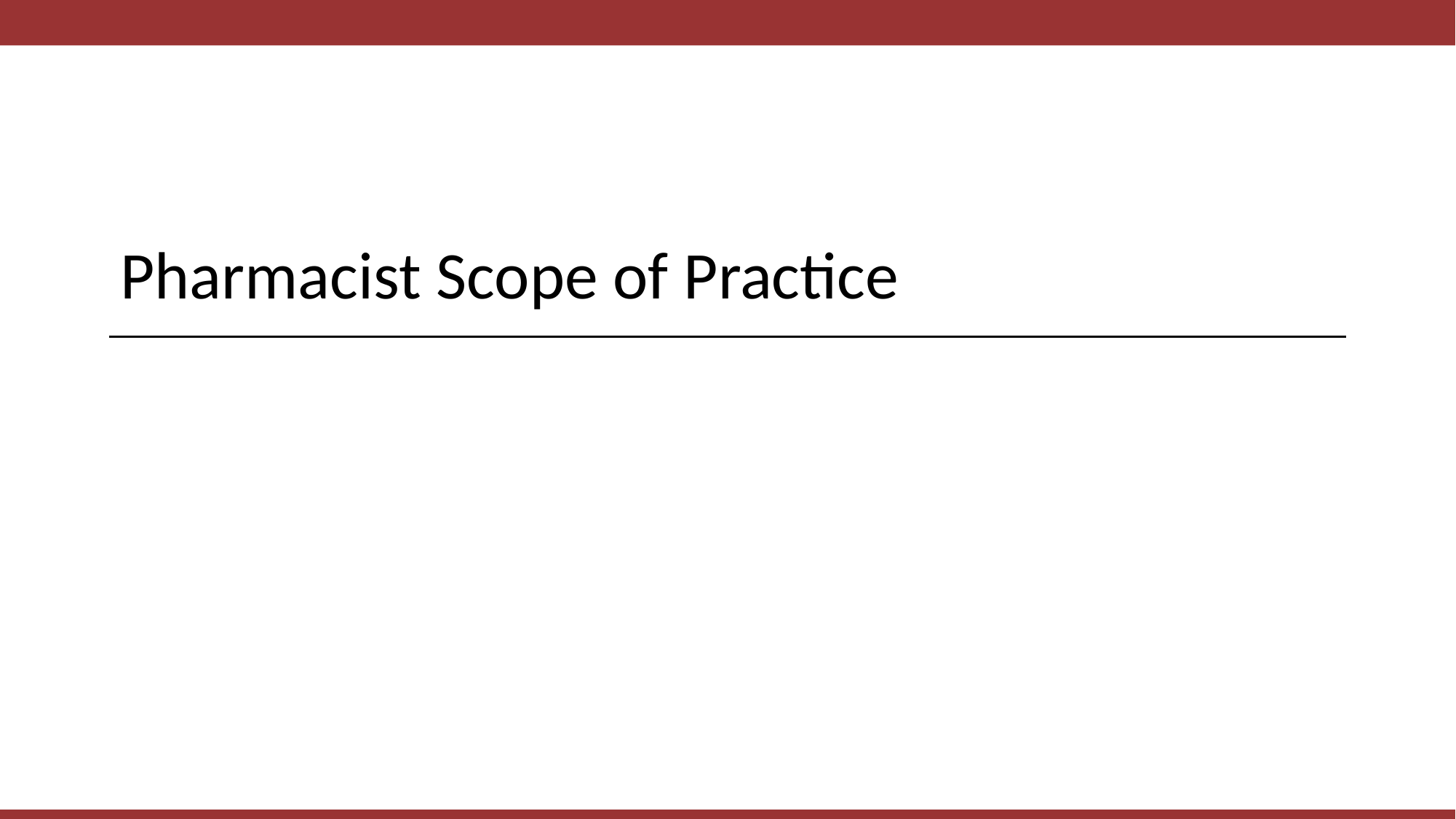

# Pharmacist Scope of Practice

## Slide 2
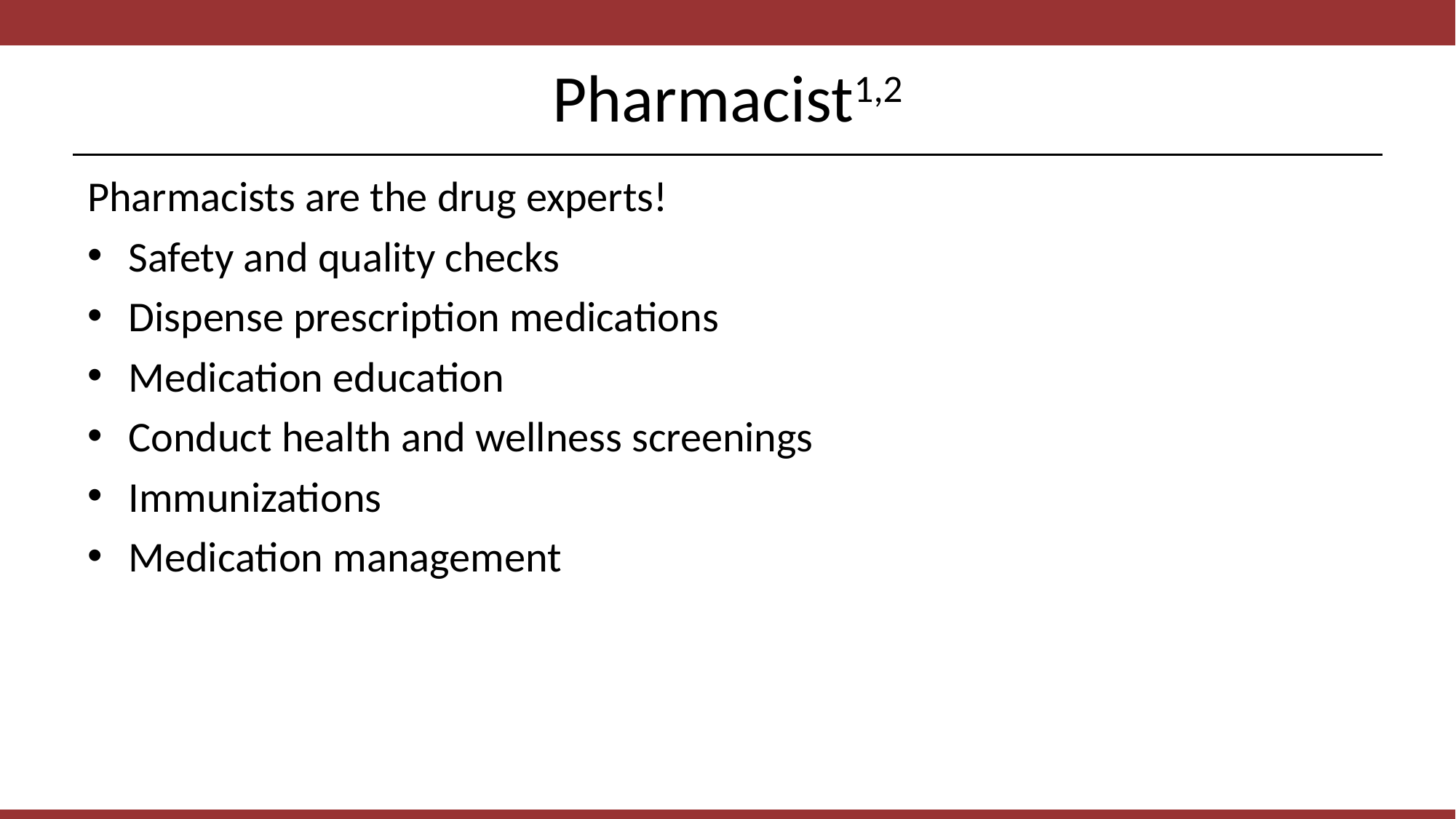

# Pharmacist1,2
Pharmacists are the drug experts!
Safety and quality checks
Dispense prescription medications
Medication education
Conduct health and wellness screenings
Immunizations
Medication management

## Slide 3
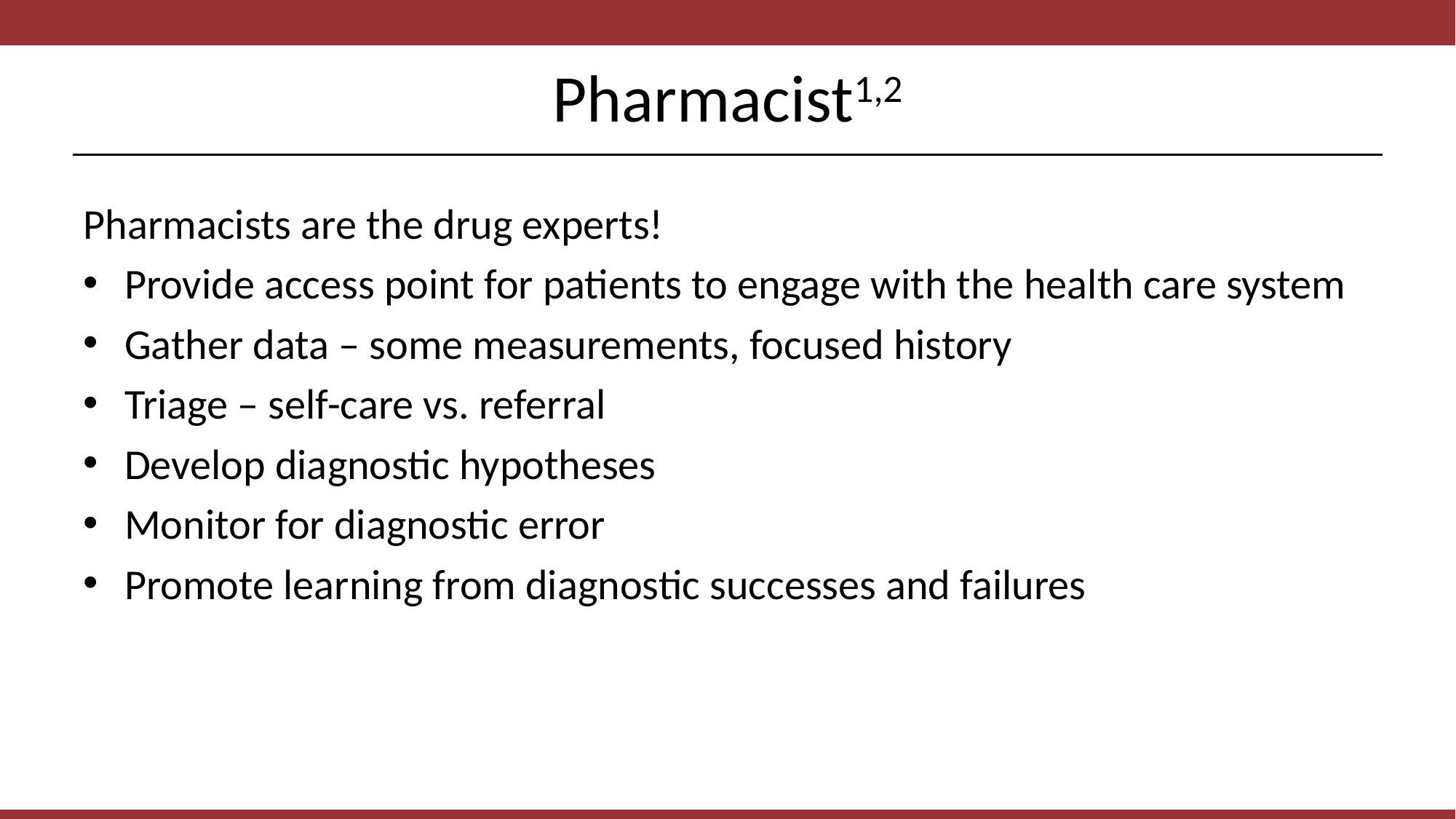

# Pharmacist1,2
Pharmacists are the drug experts!
Provide access point for patients to engage with the health care system
Gather data – some measurements, focused history
Triage – self-care vs. referral
Develop diagnostic hypotheses
Monitor for diagnostic error
Promote learning from diagnostic successes and failures

## Slide 4
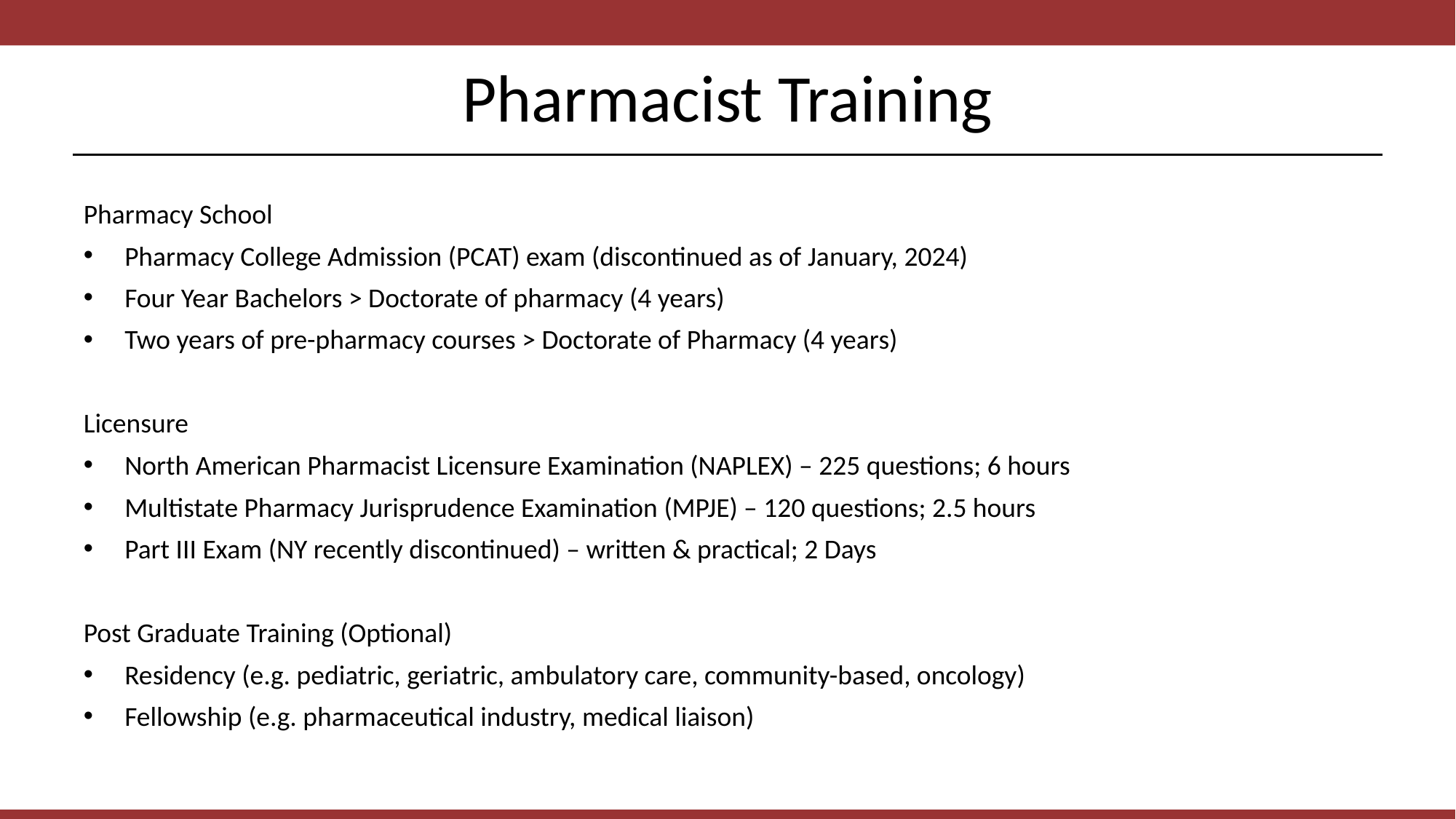

# Pharmacist Training
Pharmacy School
Pharmacy College Admission (PCAT) exam (discontinued as of January, 2024)
Four Year Bachelors > Doctorate of pharmacy (4 years)
Two years of pre-pharmacy courses > Doctorate of Pharmacy (4 years)
Licensure
North American Pharmacist Licensure Examination (NAPLEX) – 225 questions; 6 hours
Multistate Pharmacy Jurisprudence Examination (MPJE) – 120 questions; 2.5 hours
Part III Exam (NY recently discontinued) – written & practical; 2 Days
Post Graduate Training (Optional)
Residency (e.g. pediatric, geriatric, ambulatory care, community-based, oncology)
Fellowship (e.g. pharmaceutical industry, medical liaison)

## Slide 5
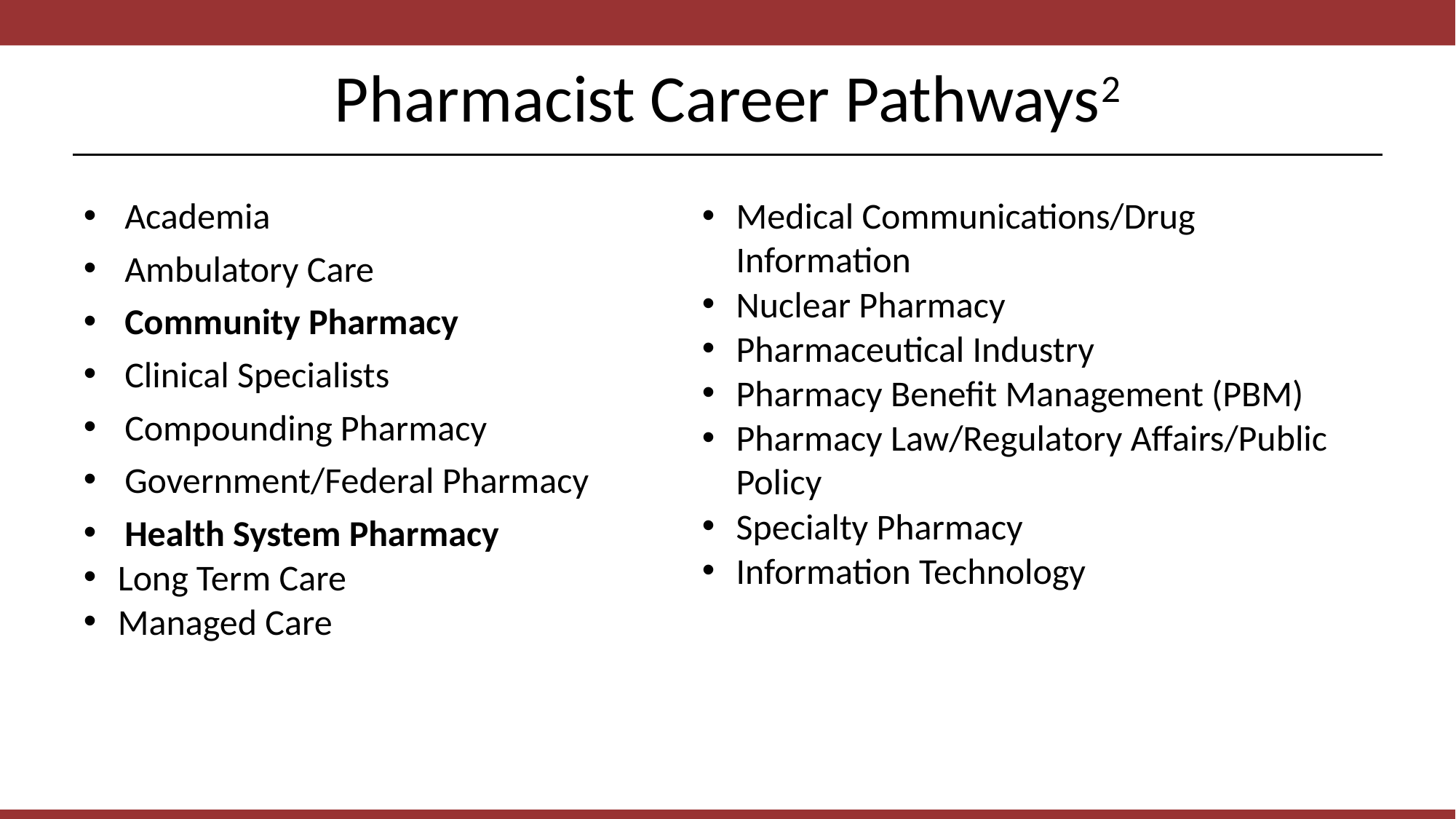

# Pharmacist Career Pathways2
Academia
Ambulatory Care
Community Pharmacy
Clinical Specialists
Compounding Pharmacy
Government/Federal Pharmacy
Health System Pharmacy
Long Term Care
Managed Care
Medical Communications/Drug Information
Nuclear Pharmacy
Pharmaceutical Industry
Pharmacy Benefit Management (PBM)
Pharmacy Law/Regulatory Affairs/Public Policy
Specialty Pharmacy
Information Technology

## Slide 6
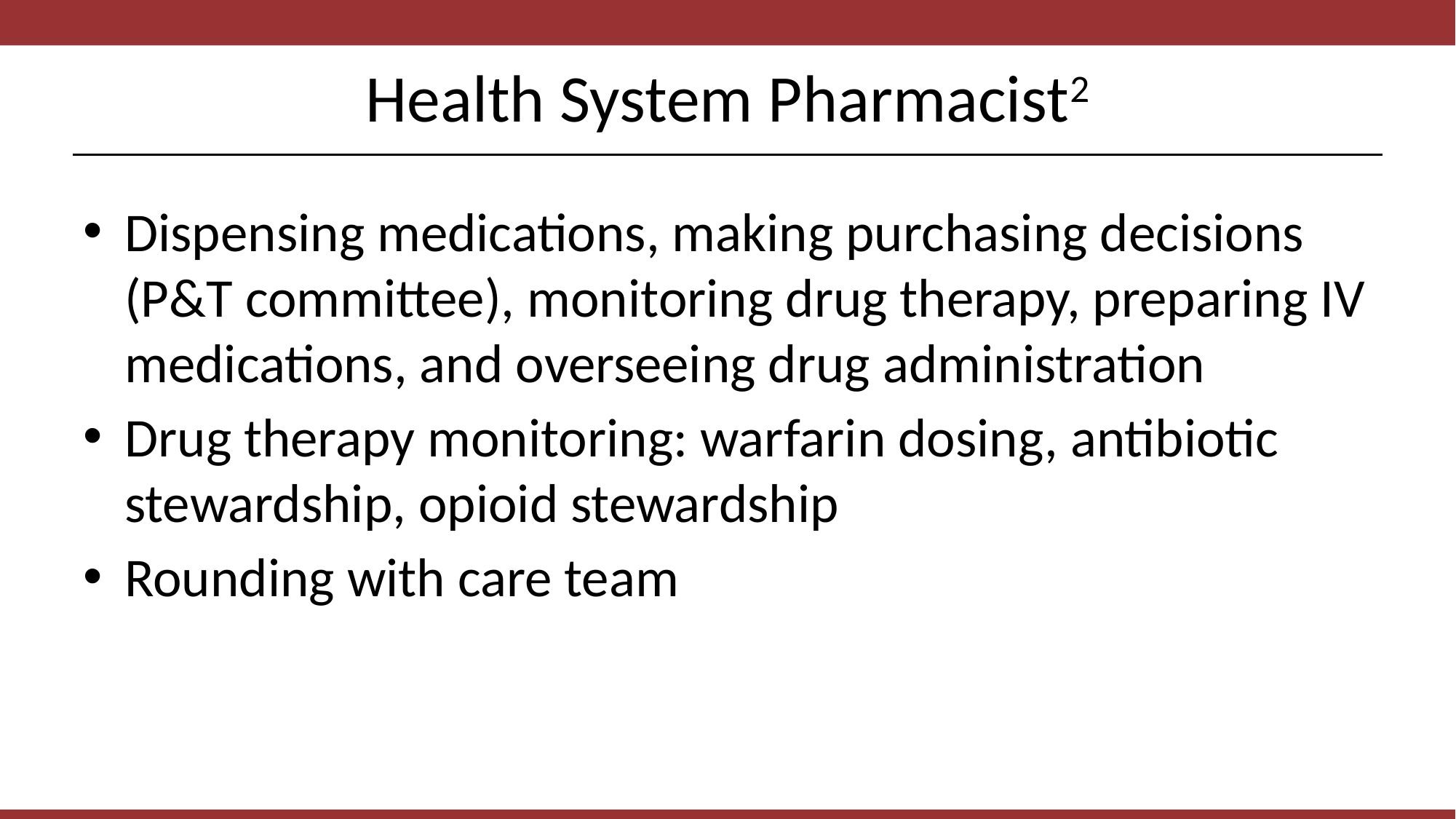

# Health System Pharmacist2
Dispensing medications, making purchasing decisions (P&T committee), monitoring drug therapy, preparing IV medications, and overseeing drug administration
Drug therapy monitoring: warfarin dosing, antibiotic stewardship, opioid stewardship
Rounding with care team

## Slide 7
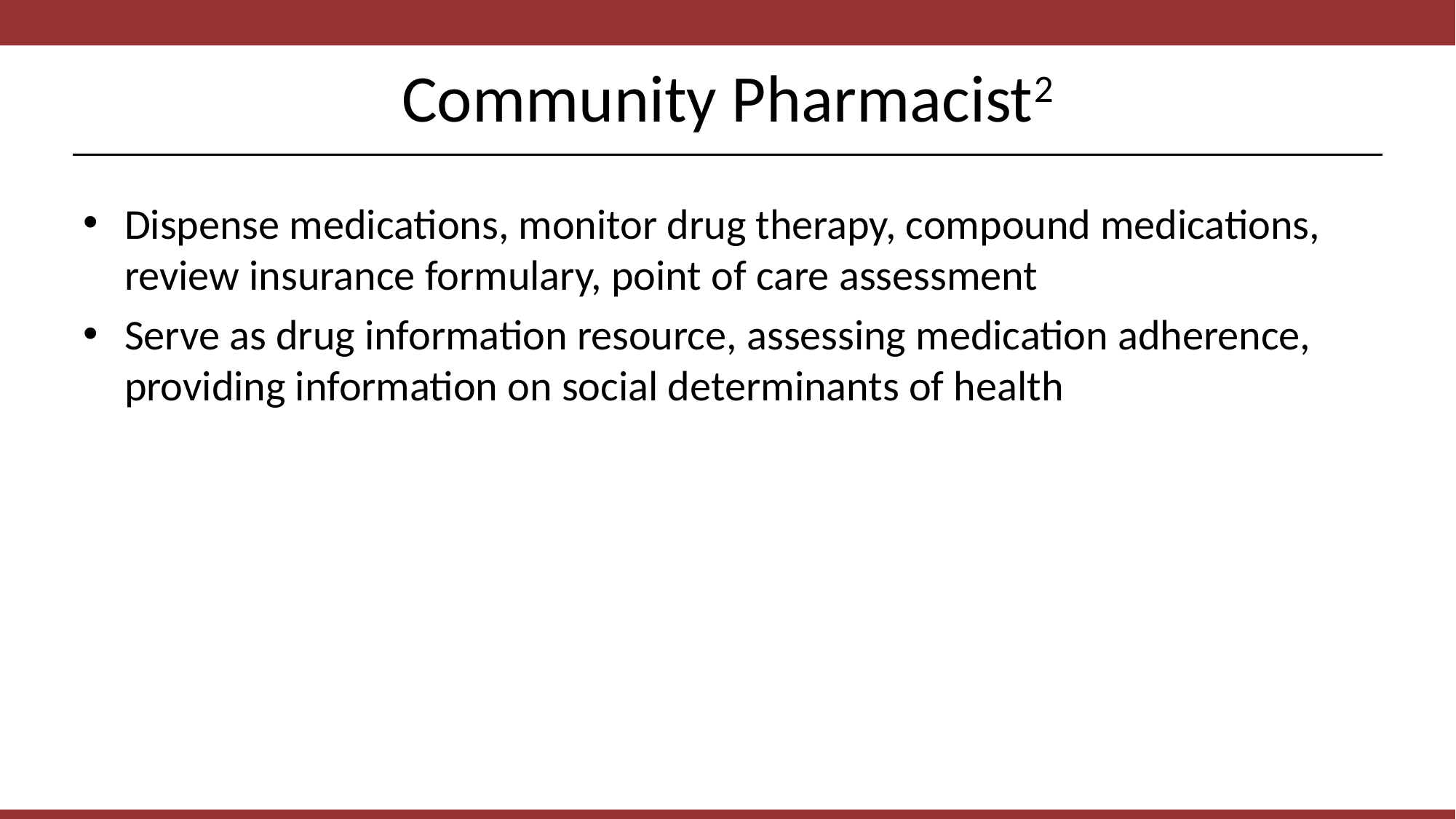

# Community Pharmacist2
Dispense medications, monitor drug therapy, compound medications, review insurance formulary, point of care assessment
Serve as drug information resource, assessing medication adherence, providing information on social determinants of health

## Slide 8
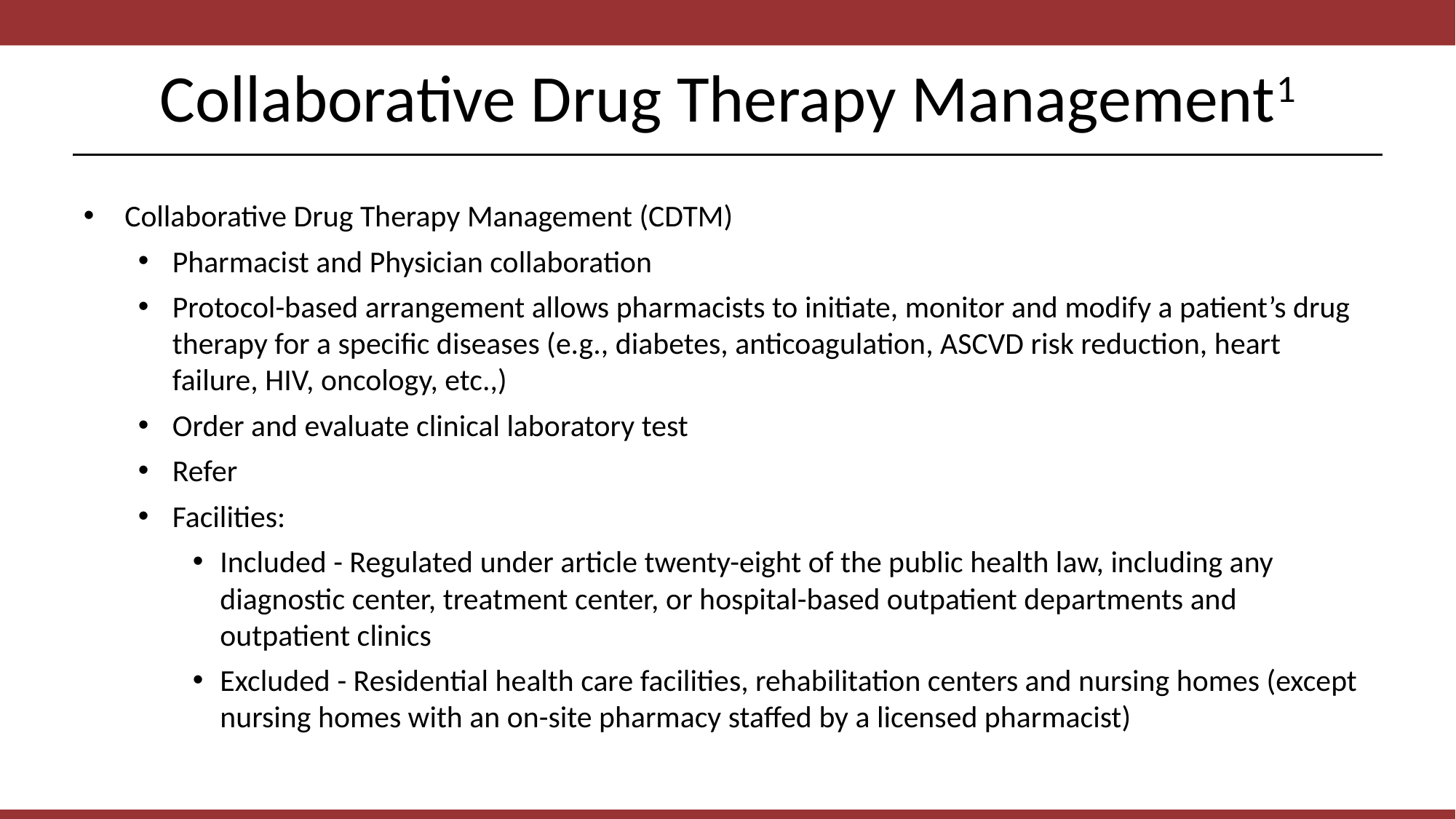

# Collaborative Drug Therapy Management1
Collaborative Drug Therapy Management (CDTM)
Pharmacist and Physician collaboration
Protocol-based arrangement allows pharmacists to initiate, monitor and modify a patient’s drug therapy for a specific diseases (e.g., diabetes, anticoagulation, ASCVD risk reduction, heart failure, HIV, oncology, etc.,)
Order and evaluate clinical laboratory test
Refer
Facilities:
Included - Regulated under article twenty-eight of the public health law, including any diagnostic center, treatment center, or hospital-based outpatient departments and outpatient clinics
Excluded - Residential health care facilities, rehabilitation centers and nursing homes (except nursing homes with an on-site pharmacy staffed by a licensed pharmacist)

## Slide 9
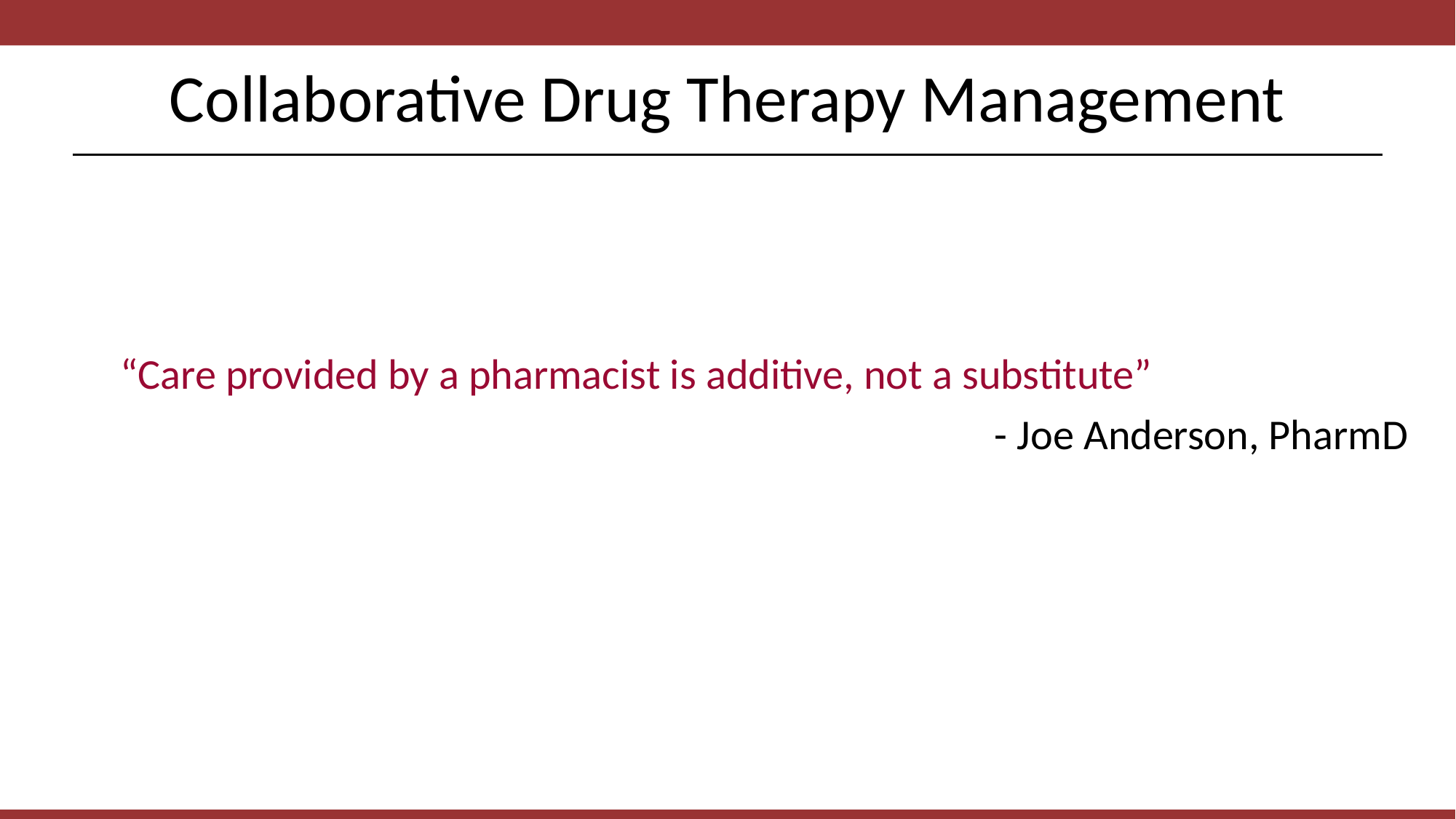

# Collaborative Drug Therapy Management
“Care provided by a pharmacist is additive, not a substitute”
- Joe Anderson, PharmD

## Slide 10
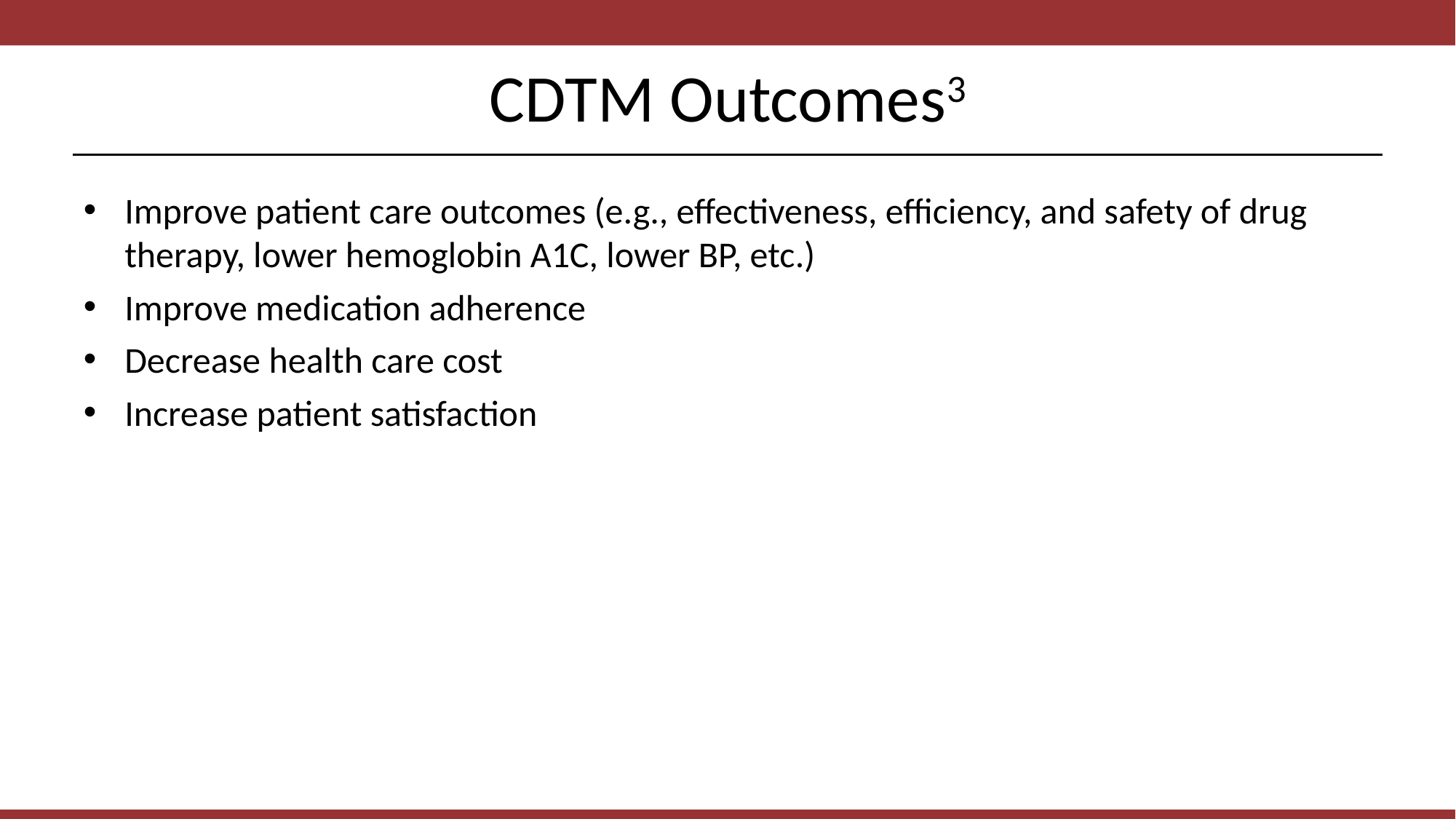

# CDTM Outcomes3
Improve patient care outcomes (e.g., effectiveness, efficiency, and safety of drug therapy, lower hemoglobin A1C, lower BP, etc.)
Improve medication adherence
Decrease health care cost
Increase patient satisfaction

## Slide 11
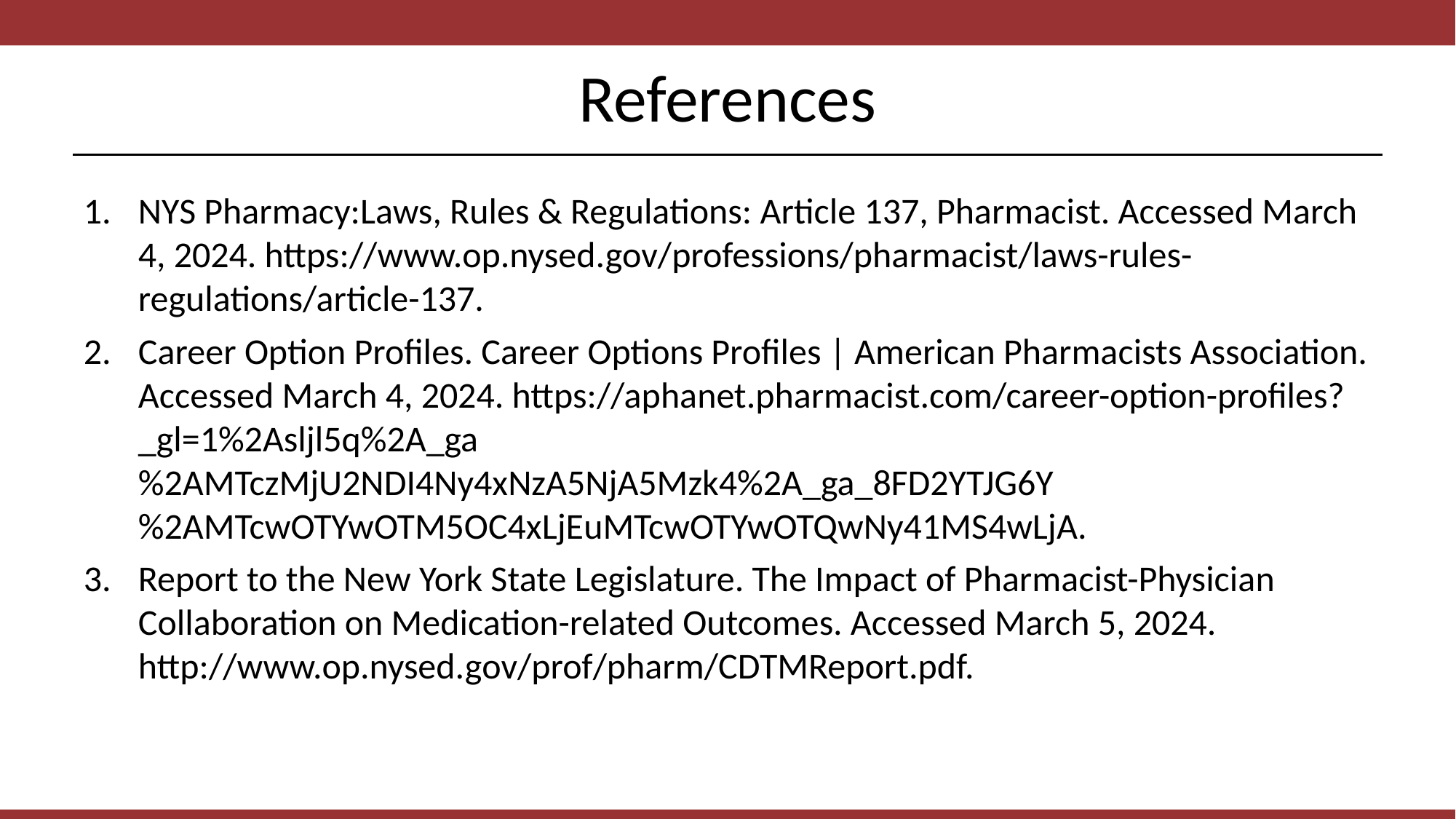

# References
NYS Pharmacy:Laws, Rules & Regulations: Article 137, Pharmacist. Accessed March 4, 2024. https://www.op.nysed.gov/professions/pharmacist/laws-rules-regulations/article-137.
Career Option Profiles. Career Options Profiles | American Pharmacists Association. Accessed March 4, 2024. https://aphanet.pharmacist.com/career-option-profiles?_gl=1%2Asljl5q%2A_ga%2AMTczMjU2NDI4Ny4xNzA5NjA5Mzk4%2A_ga_8FD2YTJG6Y%2AMTcwOTYwOTM5OC4xLjEuMTcwOTYwOTQwNy41MS4wLjA.
Report to the New York State Legislature. The Impact of Pharmacist-Physician Collaboration on Medication-related Outcomes. Accessed March 5, 2024. http://www.op.nysed.gov/prof/pharm/CDTMReport.pdf.

## Slide 12
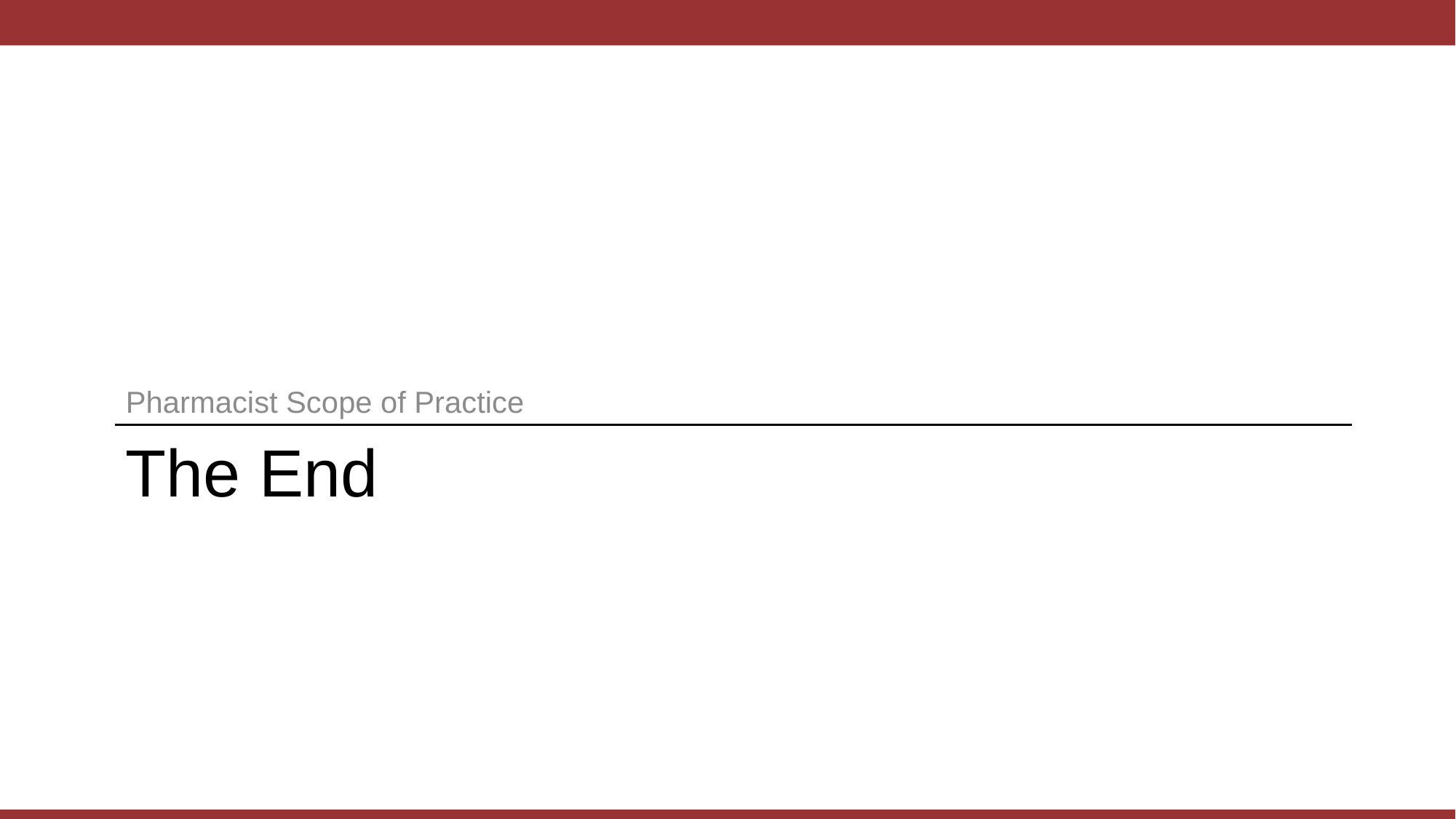

Pharmacist Scope of Practice
