# Supplementary material for: Interprofessional Coproduction of Diagnosis with Medical and Pharmacy Students: An Interactive Case-Based Workshop
Source: MedEdPORTAL. 2024 Sep 24;20:11437. doi: 10.15766/mep_2374-8265.11437 (PMC11402627; doi:10.15766/mep_2374-8265.11437)
Supplement: Supplementary file 1 — Session Outline for Students.docxIntro to Diagnostic Error and IP Dx.pptxPharmacist Scope of Practice.pptxInterprofessional Case Facilitator Guide.docxAliquot 1 for Medical Students.docxAliquot 1 for Pharmacy Students.docxAliquot 2 for Medical Students.docxAliquot 2 for Pharmacy Students.docxIndividual Reflection After Aliquot 1.docxIndividual Reflection After Aliquot 2.docxWrap-up Session Slides.pptx [file mep_2374-8265.11437-s001.zip › D. Interprofessional Case Facilitator Guide.docx]

**Facilitator Guide**

**Executive Summary**

This is a case discussion to encourage students to synthesize a case into a concise summary statement, develop a differential diagnosis, and incorporate additional information from other members of the interprofessional team into the differential diagnosis.

All case information is conveyed by word document, but is provided in two aliquots.

- **Aliquot 1 for pharmacy** **students** provides the patient’s med lists and pick-up dates, along with a phone message from her son concerned about her dizziness.
- **Aliquot 1 for med students** provides a synopsis of PMH, last office visit, and hospitalization, along with a MyChart message from her son concerned about her dizziness.
- **Aliquot 2 for pharmacy** **students** provides BP, HR, and FSBG from the visiting nurse.
- **Aliquot 2 for med students** provides the same info, plus a negative physical exam except for bradycardia.

The medical students and pharmacy students will receive information in each aliquot, but each receives only part of the case. Tell students that they will receive information that is authentic for the role in which they are playing. For medical students this information is consistent to what they will see when working in a PCP office. For pharmacy students, this information is consistent with what they will see when working in a community pharmacy.

They should talk with each other to share this information.

Do not reveal the second aliquot until the group has completed their discussion.

**Case synopsis (for facilitators only)**

**Patient: Chaarumathi Joshi**

Age: 62 years old

Female refugee from Nepal.

Speaks rudimentary English; doesn’t read in either language

She lives with her son **Amir Joshi**, who is her primary caregiver, and his wife. He is fluent in English and translates for her, because her dialect is not available with interpreter services. Amir is very attentive but has limited health literacy.

Ms. Joshi was discharged from the hospital 3 days ago, after treatment of a bleeding peptic ulcer. She now has pre-syncope. Amir sends a patient portal message to the primary care physician’s office, telling them that she is dizzy. He also leaves a message with the pharmacist, wondering if he forgot to pick up one of her medications. *Note: His suggestion of “underdosing” her medications is an intentional red herring, because in fact, she is taking too many medications.* Her medication lists from pre-hospitalization and discharge are not aligned. The pharmacy students will have some of this information and know when she had picked up her last prescriptions prior to the hospital stay.

If they talk with each other, they will learn that she is taking two beta blockers- the long-acting formulation of metoprolol, which she was taking prior to hospitalization, and the short-acting form that she was prescribed on discharge. Transitions in care (e.g. hospital to home) are high risk situations for medications. In older patients, medication duplications, omissions, and interactions, frequently cause side effects. Robust medication reconciliation, including creating a “best possible medical list (BPML)” on admission adds safety, as does communication between physician and pharmacist.

Students will generate a summary statement and a differential diagnosis.

She has multiple **chronic** conditions that could contribute to pre-syncope:

- Atrial fibrillation (if in rapid a-fib or overly blocked)
- Type 2 diabetes mellitus (if hyper- or hypo-glycemic). Note that metformin should not trigger hypoglycemia.
- Hypertension (if she took too much of her anti-hypertensive)

Students should also consider her **acute** conditions:

- She just had a GI bleed. She has black stools, which could signal a recurrent upper GI bleed, but is on oral iron, which will also make her stools black.
- She was on anticoagulation for her atrial fibrillation prior to hospitalization (apixaban), but this was stopped on admission and was not prescribed on discharge. Students should consider whether she resumed this medication anyway when she returned home. Note: it is typically safe to hold this medication for several weeks after a bleeding event, and it is restarted after she is cleared by the GI physician after discharge.
- She also didn’t eat well in the hospital, but has been eating well at home. She could have some residual hypovolemia.
- The PPI could interfere with absorption of iron, but this is more of a chronic than acute problem.

**Detailed Small Group Session Outline**

**Small Group**

***10 minutes***

**Ice-breaker:** *Have each student take a turn.*

- Introduce yourself
- Share one good thing that happened this week
- Share one thing you learned from the large group sessions today

**Aliquot 1**

***15 minutes:*** Students will read the 1^st^ aliquot to themselves. . If they see the link to the 2^nd^ aliquot, ask them to PLEASE not read it yet.

As individuals, they will develop a **summary statement,** and an initial differential diagnosis with at least 3 diagnostic hypotheses.

A summary statement is generally 1-2 sentences and has 3 components:
**Background:**Demographics (e.g. age, gender if relevant), Prior illnesses, Risk factors
**Syndrome:** the presenting problem and associated key findings
**Tempo:**  onset and course of the presenting problem

- Students should follow the link to the on-line template

**20 minutes**

Group discussion:

1. Invite one pharmacy student to share a component of their summary statement, then one med student. It is normal, even for expert diagnosticians, to generate different summary statements for the same case. However they should include the 3 components above, and include the salient information (without simply repeating the entire case).

Example of a summary statement for this case (after Aliquot 1):

“Ms. Chaaramuthi is a 62yo female with PMH notable for HTN, HFpEF, Afib (anticoagulated with a DOAC), DM2, and hyperlipidemia, recently hospitalized for an upper GI bleed, now with three days of dizziness and black stools since discharge.”

2. Ask everyone to put one diagnostic hypothesis in the Chat

3. Ask what else could be causing Ms. Joshi’s pre-syncope?

Encourage students who wish to add to the differential to do so verbally.

Even if we’re certain about a diagnosis, we should maintain a habit of asking ourselves “what else?”. At this point in this case, students should not be certain anyway!

4. Gathering additional data from interprofessional team:

Ask the med students: What information might you request from her pharmacist?

Ask the pharmacy students: What information might you request from her physician?

5. Student discussion.

Facilitator: “Okay, now I’m going to step back, and you have 10 minutes to talk with each other and think through the differential diagnosis. Feel free to ask each other questions.” Encourage students in med/pharm ask each other questions.

**Aliquot 2**

**15 minutes total**

2 minutes: Students read Aliquot 2 on their own

13 minutes: Group discussion

1. What critical information or “key findings” have you learned?

These may be abnormal findings or normal findings.

How does this info impact your differential diagnosis?

What diagnostic hypotheses can you rule out?

What’s rising to the top of the list.

1. Student discussion. Facilitator: “Okay, now I’m going to step back again, and you have **5 minutes** to talk with each other and refine your differential diagnosis. Feel free to ask each other questions.” Encourage students in med/pharm to ask each other questions.
2. Commit: “Okay. It’s time to prioritize our differential diagnosis. We may not be completely certain about the final diagnosis, but let’s identify our top 3 most likely diagnoses.” You can help get this started based on what you heard in their discussion.

You may combine # 2 and #3, depending on how the discussion flows. Note: if there’s time, you can ask what additional workup they might get, but this isn’t critical.

**5 minutes: Individual Reflection**

- All students should follow the link to the on-line template.

**On their own, they will document their responses to the following:**

How many diagnostic hypotheses did you think of on your own?

How did talking with the pharmacy/med student alter your differential diagnosis, if at all? Was there anything you learned from them that you hadn’t already considered?

**5 minutes:** **Group reflection:**

Great job! Ask for volunteers from each specialty to share their thoughts from the individual reflection.

**Student versions of the case**

*This is what appears in the students’ aliquots.*

*Do not reveal this info to the students; they should discover it on their own by reading and talking with each other.*

**Aliquot 1**

***Medical students:***

*You’re working in the outpatient office. You receive an InBasket message from the son of your patient Chaarumathi Joshi. You open her chart.*

**Patient: Chaarumathi Joshi**

Age: 62 years old

Female refugee from Nepal.

Speaks rudimentary English; doesn’t read in either language

She lives with her son **Amir Joshi**, who is her primary caregiver, and his wife. He is fluent in English and translates for her, because her dialect is not available with interpreter services. Amir is very attentive but has limited health literacy.

***MyChart message:***

My mom was discharged from the hospital three days ago. She was bleeding from her stomach, but they stopped the bleeding. Since she got home, whenever she stands up, she gets dizzy. It gets better when she sits down. She’s taking her medications, and my wife is making sure she’s eating and drinking. The hospital doctors said her stools might be dark, but I’m worried that they still look almost black.

**You review her chart.** One week ago, you saw Ms. Joshi in your office for epigastric pain, lightheadedness, and black stool.

You sent her to the hospital. She was admitted and found to have a 1cm duodenal ulcer with stigmata of recent bleeding. The ulcer was treated with a laser, and she was transfused one unit of packed red blood cells. She had no further bleeding, and was discharged home 3 days ago. Her hemoglobin at discharge was 10.5 g/dL, and her serum creatinine was 1.1 mg/dL. She was instructed to hold apixaban, torsemide, and amlodipine until a follow-up visit. She is scheduled to follow up with gastroenterology about the results of her *Helicobacter pylori* test.

**PMH**

Hypertension

Heart failure with preserved ejection fraction (HFpEF)

Atrial fibrillation

Hyperlipidemia

Peptic ulcer disease

Iron deficiency anemia

Type 2 diabetes mellitus

**Medications at last office visit**

Metoprolol succinate 50 mg PO daily

Torsemide 20 mg PO daily

Amlodipine 5 mg PO daily

Rosuvastatin 10 mg PO daily

Apixaban 5 mg PO BID

Metformin 500 mg PO daily

**Social History**

From rural Nepal. Emigrated 8 years ago.

Lives with son and his wife and 3 children. Her son functions as her translator (translation services don’t include her dialect).

No history of alcohol, tobacco, or other substance use.

Helps around the house, cooking, cleaning.

*You call her son, but he’s not answering the phone.*

**Next Step:** Open the link to the template and generate a summary statement and a differential diagnosis with the information you have so far. You should do this independently, before you start talking with your group.

**Aliquot 1**

***Pharmacy Students***

You’re working at the local pharmacy when a regular customer (Amir Joshi) calls about his mother’s medications. He picked up Chaaramuthi’s medications when she was discharged from the hospital 3 days ago. He set up her medications, and tells you she’s been taking them regularly, but for the past 3 days she’s been feeling dizzy. He wonders if all of her medications were included with the last pick-up, or whether one was missed or underdosed.

You’ve spoken with Mr. Joshi several times before. His family immigrated from Nepal, and you’ve admired how caring he is with his mother. He often brings his mother with him to check her blood pressure with the store’s machine. Since the pandemic, you haven’t seen her at all, but you recall that she doesn’t speak English, and he translated for her. He always picks up her medications on time. Her dispense record for the last 60 days is below.

**Meds**

| **Date dispensed** | **Medication** | **Directions** | **Quantity** | **Prescriber** |
| --- | --- | --- | --- | --- |
| **T-19 days** | **Amlodipine 5 mg tablet** | **1 tab PO daily** | **30** | **Dr. Brown** |
| **T-39 days** |  |  |  |  |
| **T-19 days** | **Apixaban 5 mg tablet** | **1 tab PO BID** | **60** | **Dr. Brown** |
| **T-39 days** |  |  |  |  |
| **T-3 days** | **Ferrous sulfate 325 mg tablet** | **1 tab PO every other day** | **15** | **Dr. Thomas** |
| **T-19 days** | **Metformin HCl 500 mg ER tablet** | **1 tab PO daily** | **30** | **Dr. Brown** |
| **T-39 days** |  |  |  |  |
| **T-19 days** | **Metoprolol succinate 50 mg ER tablet** | **1 tab PO daily** | **30** | **Dr. Brown** |
| **T-39 days** |  |  |  |  |
| **T-3 days** | **Metoprolol tartrate 25 mg tablet** | **1 tab PO BID** | **60** | **Dr. Thomas** |
| **T-3 days** | **Pantoprazole 40 mg EC tablet** | **1 tab PO daily** | **30** | **Dr. Brown** |
| **T-19 days** | **Rosuvastatin 10 mg tablet** | **1 tab PO daily** | **30** | **Dr. Brown** |
| **T-39 days** |  |  |  |  |
| **T-19 days** | **Torsemide 20 mg tablet** | **1 tab PO daily** | **30** | **Dr. Brown** |
| **T-39 days** |  |  |  |  |

**Discontinue metoprolol succinate received 3 days ago from Dr. Thomas.**

**Next step:** Open the link to the on-line template and summarize the case and identify 3 diagnostic hypotheses.

**Aliquot 2**

*Med students:*

Prior to discharge from the hospital, the inpatient team arranged for a visiting nurse to conduct daily visits in Ms. Chaaramuthi’s home.

You ask the visiting nurse to call your office after they do their assessment today.

The nurse confirms the history that the son provided, and notes that Ms. Joshi’s lungs are clear, and she has no swelling in her legs. Her heart rate is irregular and slow with no murmurs. Her abdominal exam is normal.

Her BP is 110/72 (sitting) and her heart rate is 48.

Her finger stick blood glucose is 118.

**Note:** At the end of the group discussion of this aliquot, follow the link and complete the brief reflection.

**Aliquot 2**

*Pharmacy students:*

Amir tells you that the visiting nurse was at the house and took his mom’s vital signs.

Her BP is 110/72 mm Hg (sitting) and her heart rate is 48.

Her finger stick blood glucose is 118 mg/dL.

Her weight is 65.2 kg.

After you ask, Amir tells you that his mother has been taking all of her medications since discharge, including the new ones. She never misses doses, because he fills a weekly pillbox for her every Sunday and then he or his wife uses it throughout the week to administer her medications. She takes all of her meds in the morning after breakfast and then if there is a second dose, she takes it after dinner.

**Note:** After you’ve discussed this aliquot with your small group, click on thelink to complete a brief reflection.
