# Supplementary material for: Interprofessional Coproduction of Diagnosis with Medical and Pharmacy Students: An Interactive Case-Based Workshop
Source: MedEdPORTAL. 2024 Sep 24;20:11437. doi: 10.15766/mep_2374-8265.11437 (PMC11402627; doi:10.15766/mep_2374-8265.11437)
Supplement: Supplementary file 1 — Session Outline for Students.docxIntro to Diagnostic Error and IP Dx.pptxPharmacist Scope of Practice.pptxInterprofessional Case Facilitator Guide.docxAliquot 1 for Medical Students.docxAliquot 1 for Pharmacy Students.docxAliquot 2 for Medical Students.docxAliquot 2 for Pharmacy Students.docxIndividual Reflection After Aliquot 1.docxIndividual Reflection After Aliquot 2.docxWrap-up Session Slides.pptx [file mep_2374-8265.11437-s001.zip › E. Aliquot 1 for Medical Students.docx]

**Aliquot 1**

*You’re working in the outpatient office. You receive an InBasket message from the son of your patient Chaarumathi Joshi. You open her chart.*

**Patient: Chaarumathi Joshi**

Age: 62 years old

Female refugee from Nepal.

Speaks rudimentary English; doesn’t read in either language

She lives with her son **Amir Joshi**, who is her primary caregiver, and his wife. He is fluent in English and translates for her, because her dialect is not available with interpreter services. Amir is very attentive but has limited health literacy.

***MyChart message:***

My mom was discharged from the hospital three days ago. She was bleeding from her stomach, but they stopped the bleeding. Since she got home, whenever she stands up, she gets dizzy. It gets better when she sits down. She’s taking her medications, and my wife is making sure she’s eating and drinking. The hospital doctors said her stools might be dark, but I’m worried that they still look almost black.

**You review her chart.** One week ago, you saw Ms. Joshi in your office for epigastric pain, lightheadedness, and black stool. You sent her to the hospital. She was admitted and found to have a 1cm duodenal ulcer with stigmata of recent bleeding. The ulcer was treated with a laser, and she was transfused one unit of packed red blood cells. She had no further bleeding, and was discharged home 3 days ago. Her hemoglobin at discharge was 10.5 g/dL, and her serum creatinine was 1.1 mg/dL. She was instructed to hold apixaban, torsemide, and amlodipine until a follow-up visit. She is scheduled to follow up with gastroenterology about the results of her *Helicobacter pylori* test.

**PMH**

Hypertension

Heart failure with preserved ejection fraction (HFpEF)

Atrial fibrillation

Hyperlipidemia

Peptic ulcer disease

Iron deficiency anemia

Type 2 diabetes mellitus

**Medications at last office visit**

Metoprolol succinate 50 mg PO daily

Torsemide 20 mg PO daily

Amlodipine 5 mg PO daily

Rosuvastatin 10 mg PO daily

Apixaban 5 mg PO BID

Metformin 500 mg PO daily

**Social History**

From rural Nepal. Emigrated 8 years ago.

Lives with son and his wife and 3 children. Her son functions as her translator (translation services don’t include her dialect).

No history of alcohol, tobacco, or other substance use.

Helps around the house, cooking, cleaning.

*You call her son, but he’s not answering the phone.*

**Next Step:** Open the link to the template and generate a summary statement and a differential diagnosis with the information you have so far. You should do this independently, before you start talking with your group.
