# Supplementary material for: Interprofessional Coproduction of Diagnosis with Medical and Pharmacy Students: An Interactive Case-Based Workshop
Source: MedEdPORTAL. 2024 Sep 24;20:11437. doi: 10.15766/mep_2374-8265.11437 (PMC11402627; doi:10.15766/mep_2374-8265.11437)
Supplement: Supplementary file 1 — Session Outline for Students.docxIntro to Diagnostic Error and IP Dx.pptxPharmacist Scope of Practice.pptxInterprofessional Case Facilitator Guide.docxAliquot 1 for Medical Students.docxAliquot 1 for Pharmacy Students.docxAliquot 2 for Medical Students.docxAliquot 2 for Pharmacy Students.docxIndividual Reflection After Aliquot 1.docxIndividual Reflection After Aliquot 2.docxWrap-up Session Slides.pptx [file mep_2374-8265.11437-s001.zip › F. Aliquot 1 for Pharmacy Students.docx]

**Aliquot 1**

You’re working at the local pharmacy when a regular customer (Amir Joshi) calls about his mother’s medications. He picked up Chaaramuthi’s medications when she was discharged from the hospital 3 days ago. He set up her medications, and tells you she’s been taking them regularly, but for the past 3 days she’s been feeling dizzy. He wonders if all of her medications were included with the last pick-up, or whether one was missed or underdosed.

You’ve spoken with Mr. Joshi several times before. His family immigrated from Nepal, and you’ve admired how caring he is with his mother. He often brings his mother with him to check her blood pressure with the store’s machine. Since the pandemic, you haven’t seen her at all, but you recall that she doesn’t speak English, and he translated for her. He always picks up her medications on time. Her dispense record for the last 60 days is below.

**Meds**

| **Date dispensed** | **Medication** | **Directions** | **Quantity** | **Prescriber** |
| --- | --- | --- | --- | --- |
| **T-19 days** | **Amlodipine 5 mg tablet** | **1 tab PO daily** | **30** | **Dr. Brown** |
| **T-39 days** |  |  |  |  |
| **T-19 days** | **Apixaban 5 mg tablet** | **1 tab PO BID** | **60** | **Dr. Brown** |
| **T-39 days** |  |  |  |  |
| **T-3 days** | **Ferrous sulfate 325 mg tablet** | **1 tab PO every other day** | **15** | **Dr. Thomas** |
| **T-19 days** | **Metformin HCl 500 mg ER tablet** | **1 tab PO daily** | **30** | **Dr. Brown** |
| **T-39 days** |  |  |  |  |
| **T-19 days** | **Metoprolol succinate 50 mg ER tablet** | **1 tab PO daily** | **30** | **Dr. Brown** |
| **T-39 days** |  |  |  |  |
| **T-3 days** | **Metoprolol tartrate 25 mg tablet** | **1 tab PO BID** | **60** | **Dr. Thomas** |
| **T-3 days** | **Pantoprazole 40 mg EC tablet** | **1 tab PO daily** | **30** | **Dr. Brown** |
| **T-19 days** | **Rosuvastatin 10 mg tablet** | **1 tab PO daily** | **30** | **Dr. Brown** |
| **T-39 days** |  |  |  |  |
| **T-19 days** | **Torsemide 20 mg tablet** | **1 tab PO daily** | **30** | **Dr. Brown** |
| **T-39 days** |  |  |  |  |

**Discontinue metoprolol succinate received 3 days ago from Dr. Thomas.**

**Next step:** Open the link to the on-line template and summarize the case and identify 3 diagnostic hypotheses.
