# Supplementary material for: Interprofessional Coproduction of Diagnosis with Medical and Pharmacy Students: An Interactive Case-Based Workshop
Source: MedEdPORTAL. 2024 Sep 24;20:11437. doi: 10.15766/mep_2374-8265.11437 (PMC11402627; doi:10.15766/mep_2374-8265.11437)
Supplement: Supplementary file 1 — Session Outline for Students.docxIntro to Diagnostic Error and IP Dx.pptxPharmacist Scope of Practice.pptxInterprofessional Case Facilitator Guide.docxAliquot 1 for Medical Students.docxAliquot 1 for Pharmacy Students.docxAliquot 2 for Medical Students.docxAliquot 2 for Pharmacy Students.docxIndividual Reflection After Aliquot 1.docxIndividual Reflection After Aliquot 2.docxWrap-up Session Slides.pptx [file mep_2374-8265.11437-s001.zip › G. Aliquot 2 for Medical Students.docx]

**Aliquot 2**

Prior to discharge from the hospital, the inpatient team arranged for a visiting nurse to conduct daily visits in Ms. Chaaramuthi’s home. You ask the visiting nurse to call your office after they do their assessment today.

The nurse confirms the history that the son provided, and notes that Ms. Joshi’s lungs are clear, and she has no swelling in her legs. Her heart rate is irregular and slow with no murmurs. Her abdominal exam is normal.

Her BP is 110/72 (sitting) and her heart rate is 48.

Her finger stick blood glucose is 118.

**Note:** At the end of the group discussion of this aliquot, complete your brief reflection in the on-line template.
