# Supplementary material for: Interprofessional Coproduction of Diagnosis with Medical and Pharmacy Students: An Interactive Case-Based Workshop
Source: MedEdPORTAL. 2024 Sep 24;20:11437. doi: 10.15766/mep_2374-8265.11437 (PMC11402627; doi:10.15766/mep_2374-8265.11437)
Supplement: Supplementary file 1 — Session Outline for Students.docxIntro to Diagnostic Error and IP Dx.pptxPharmacist Scope of Practice.pptxInterprofessional Case Facilitator Guide.docxAliquot 1 for Medical Students.docxAliquot 1 for Pharmacy Students.docxAliquot 2 for Medical Students.docxAliquot 2 for Pharmacy Students.docxIndividual Reflection After Aliquot 1.docxIndividual Reflection After Aliquot 2.docxWrap-up Session Slides.pptx [file mep_2374-8265.11437-s001.zip › H. Aliquot 2 for Pharmacy Students.docx]

**Aliquot 2**

Amir tells you that the visiting nurse was at the house and took his mom’s vital signs.

Her BP is 110/72 mm Hg (sitting) and her heart rate is 48.

Her finger stick blood glucose is 118 mg/dL.

Her weight is 65.2 kg.

After you ask, Amir tells you that his mother has been taking all of her medications since discharge, including the new ones. She never misses doses, because he fills a weekly pillbox for her every Sunday and then he or his wife uses it throughout the week to administer her medications. She takes all of her meds in the morning after breakfast and then if there is a second dose, she takes it after dinner.

**Note:** After you’ve discussed this aliquot with your small group, complete your reflection in the on-line template.
