# Supplementary material for: Interprofessional Coproduction of Diagnosis with Medical and Pharmacy Students: An Interactive Case-Based Workshop
Source: MedEdPORTAL. 2024 Sep 24;20:11437. doi: 10.15766/mep_2374-8265.11437 (PMC11402627; doi:10.15766/mep_2374-8265.11437)
Supplement: Supplementary file 1 — Session Outline for Students.docxIntro to Diagnostic Error and IP Dx.pptxPharmacist Scope of Practice.pptxInterprofessional Case Facilitator Guide.docxAliquot 1 for Medical Students.docxAliquot 1 for Pharmacy Students.docxAliquot 2 for Medical Students.docxAliquot 2 for Pharmacy Students.docxIndividual Reflection After Aliquot 1.docxIndividual Reflection After Aliquot 2.docxWrap-up Session Slides.pptx [file mep_2374-8265.11437-s001.zip › I. Individual Reflection After Aliquot 1.docx]

**Individual reflection after Aliquot 1**:

1.    ​Generate a **summary statement**based on the information you have so far, understanding that you have incomplete information about the case.

A summary statement is generally 1-2 sentences and has 3 components:
**Background:**Demographics (e.g. age, gender if relevant), prior illnesses, risk factors
**Syndrome:** The presenting problem and associated key findings
**Tempo:**  Onset and course of the presenting problem

2.    What do you think might be causing her presenting problem? Identify 3 diagnostic hypotheses.

​
