# Supplementary material for: Interprofessional Coproduction of Diagnosis with Medical and Pharmacy Students: An Interactive Case-Based Workshop
Source: MedEdPORTAL. 2024 Sep 24;20:11437. doi: 10.15766/mep_2374-8265.11437 (PMC11402627; doi:10.15766/mep_2374-8265.11437)
Supplement: Supplementary file 1 — Session Outline for Students.docxIntro to Diagnostic Error and IP Dx.pptxPharmacist Scope of Practice.pptxInterprofessional Case Facilitator Guide.docxAliquot 1 for Medical Students.docxAliquot 1 for Pharmacy Students.docxAliquot 2 for Medical Students.docxAliquot 2 for Pharmacy Students.docxIndividual Reflection After Aliquot 1.docxIndividual Reflection After Aliquot 2.docxWrap-up Session Slides.pptx [file mep_2374-8265.11437-s001.zip › K. Wrap-up Session Slides.pptx]

## Slide 1
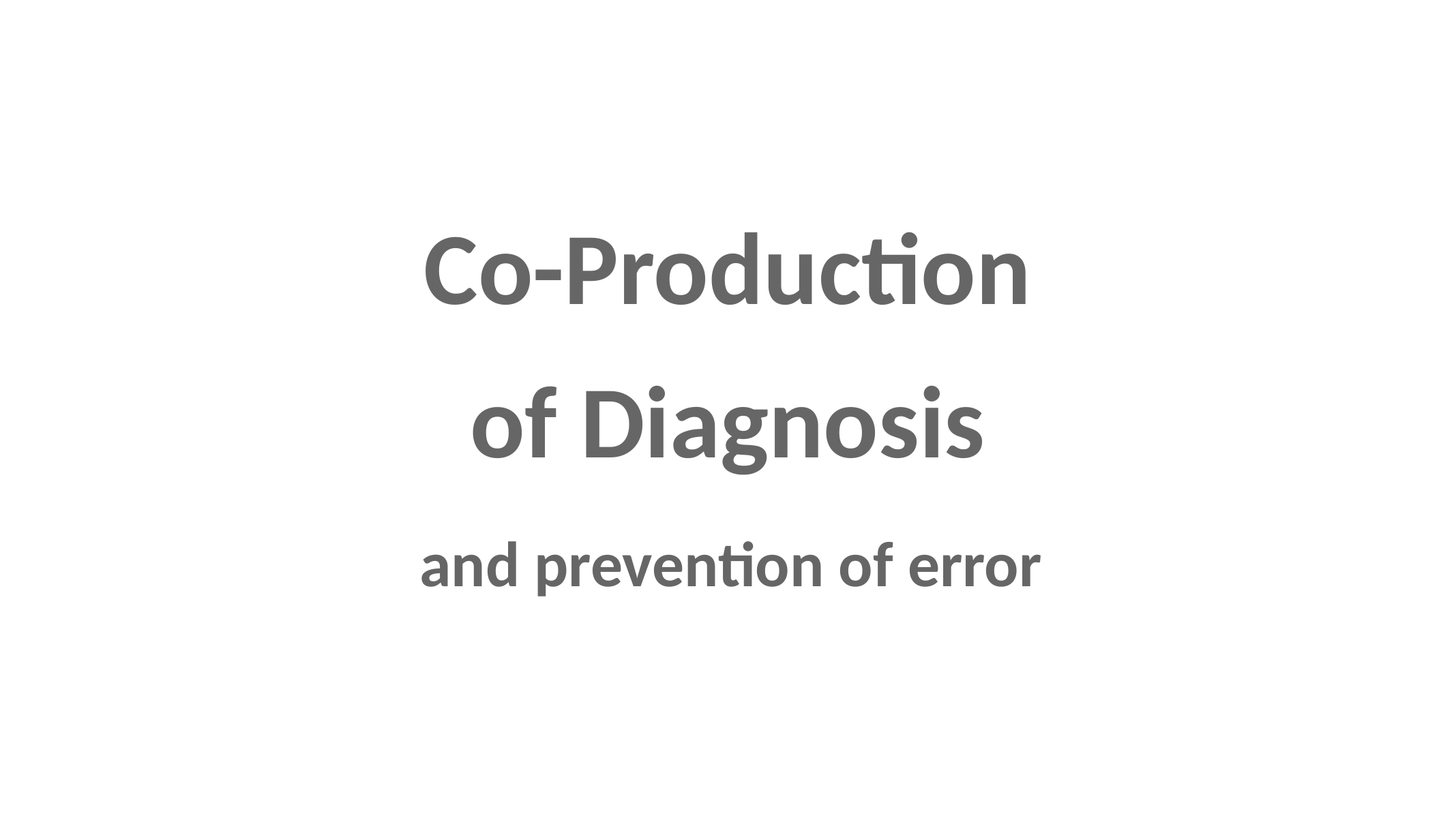

Co-Production of Diagnosis
and prevention of error

## Slide 2
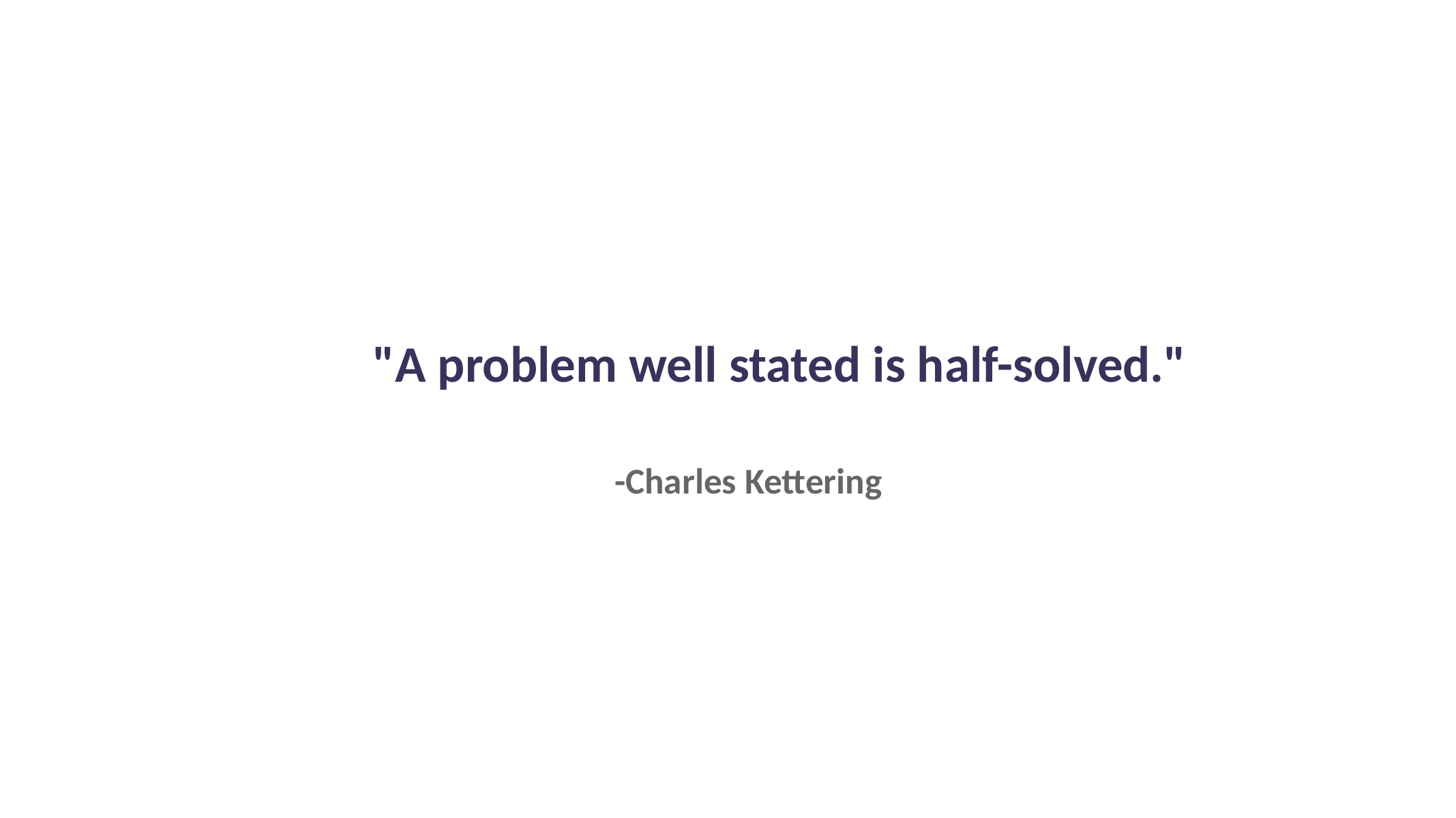

"A problem well stated is half-solved."
-Charles Kettering

## Slide 3
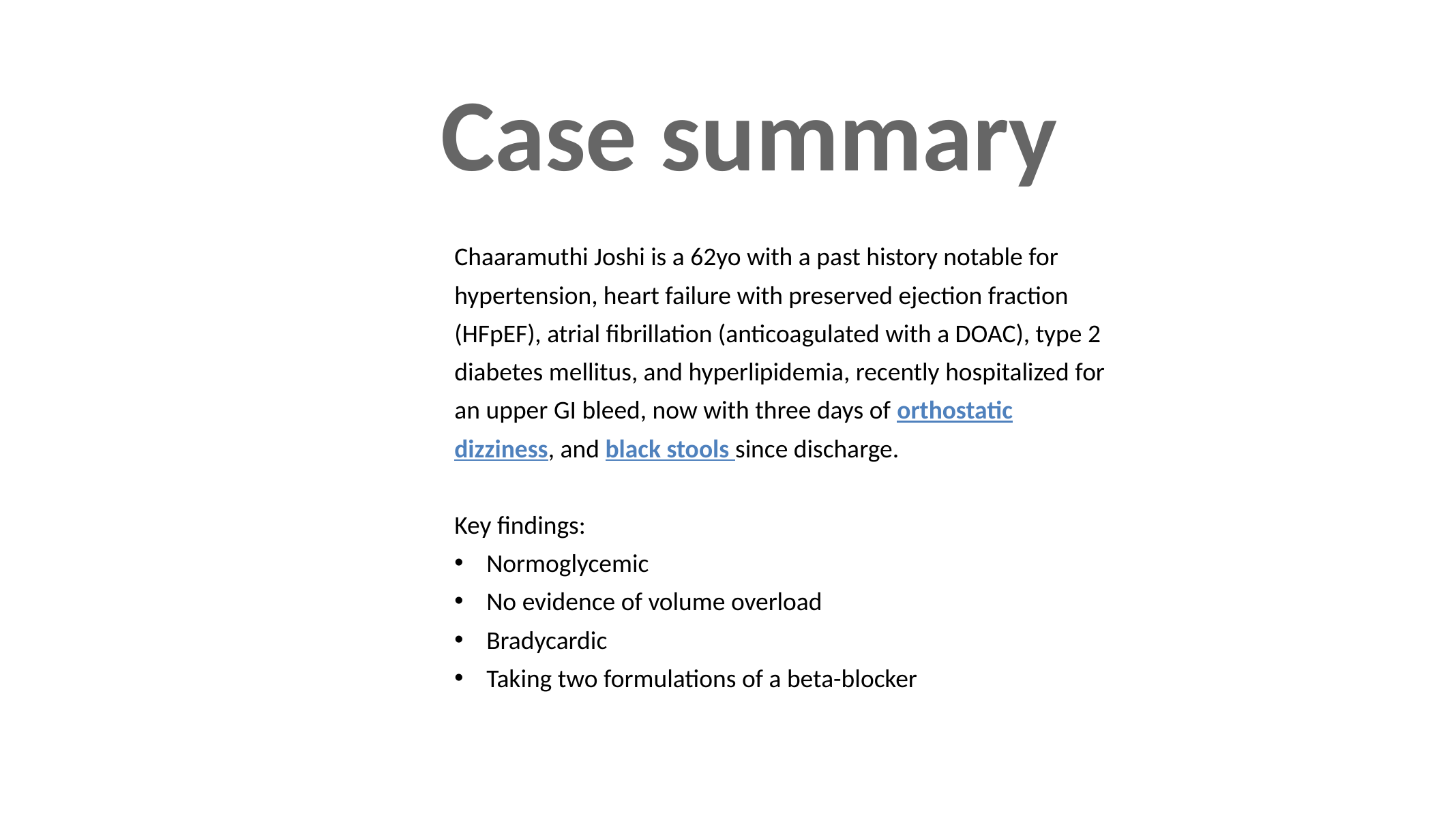

Case summary
Chaaramuthi Joshi is a 62yo with a past history notable for hypertension, heart failure with preserved ejection fraction (HFpEF), atrial fibrillation (anticoagulated with a DOAC), type 2 diabetes mellitus, and hyperlipidemia, recently hospitalized for an upper GI bleed, now with three days of orthostatic dizziness, and black stools since discharge.
Key findings:
Normoglycemic
No evidence of volume overload
Bradycardic
Taking two formulations of a beta-blocker

## Slide 4
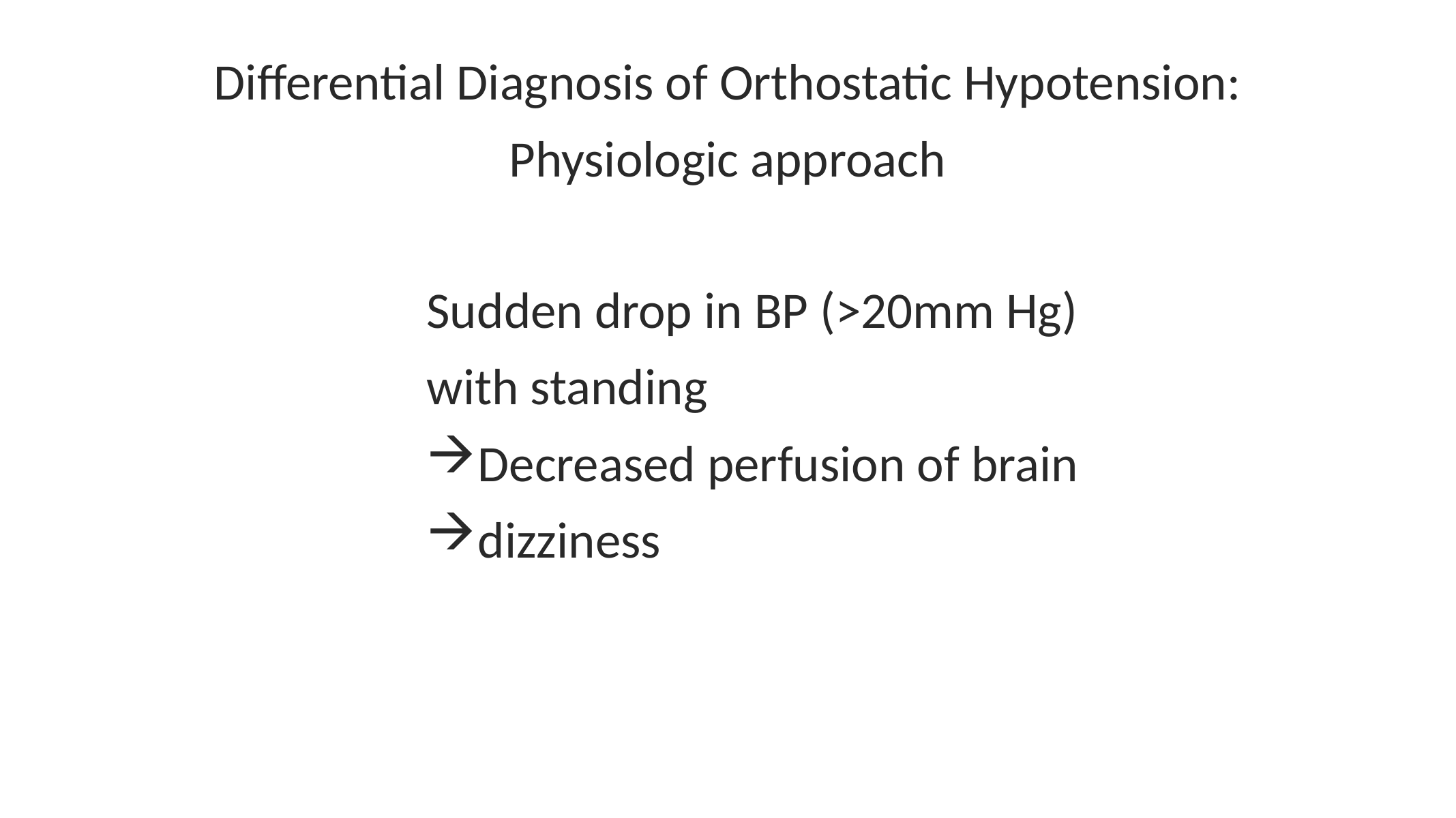

Differential Diagnosis of Orthostatic Hypotension:
Physiologic approach
Sudden drop in BP (>20mm Hg) with standing
Decreased perfusion of brain
dizziness

## Slide 5
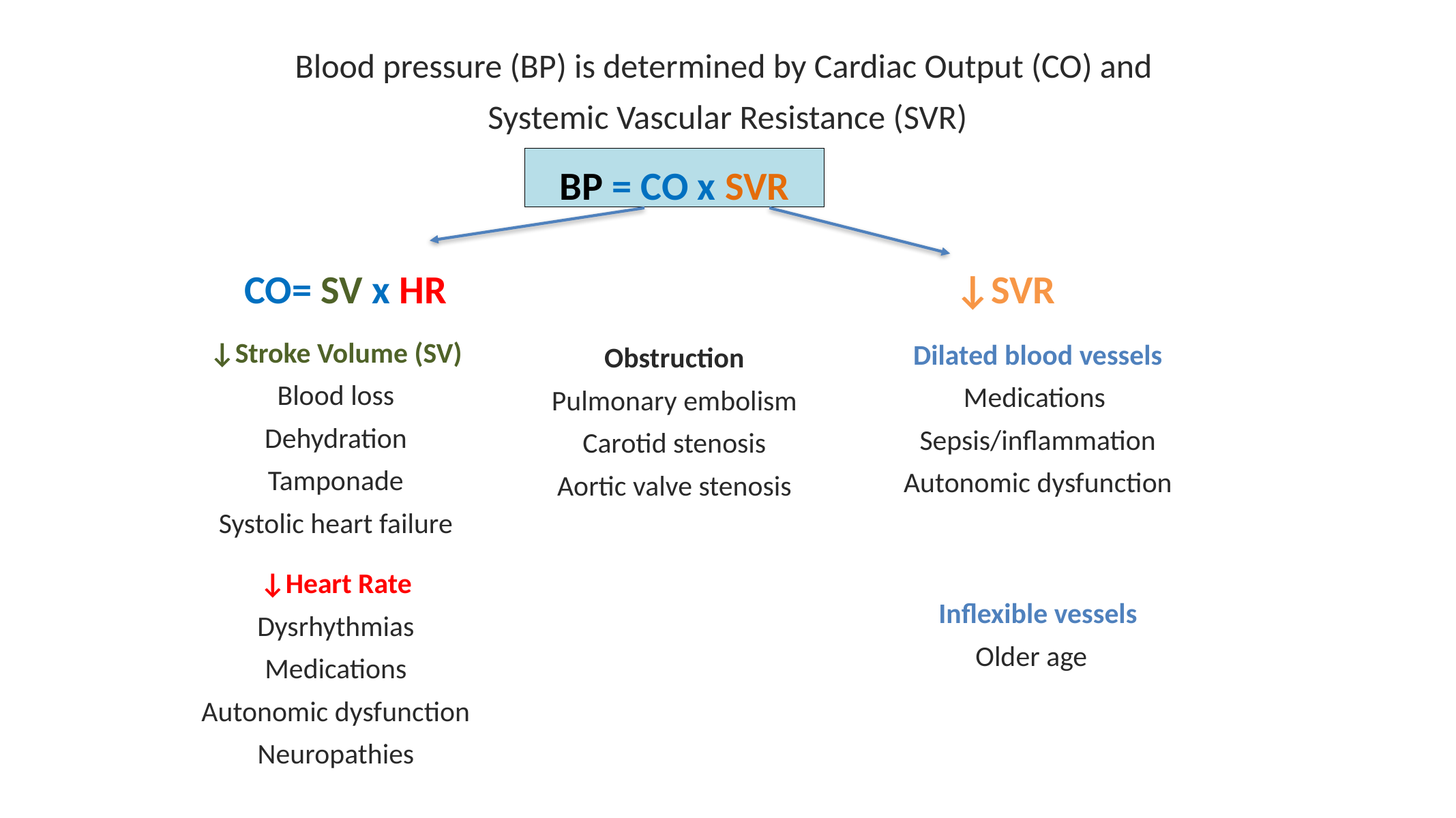

Blood pressure (BP) is determined by Cardiac Output (CO) and
Systemic Vascular Resistance (SVR)
BP = CO x SVR
CO= SV x HR
↓SVR
↓Stroke Volume (SV)
Blood loss
Dehydration
Tamponade
Systolic heart failure
Dilated blood vessels
Medications
Sepsis/inflammation
Autonomic dysfunction
Obstruction
Pulmonary embolism
Carotid stenosis
Aortic valve stenosis
↓Heart Rate
Dysrhythmias
Medications
Autonomic dysfunction
Neuropathies
Inflexible vessels
Older age

## Slide 6
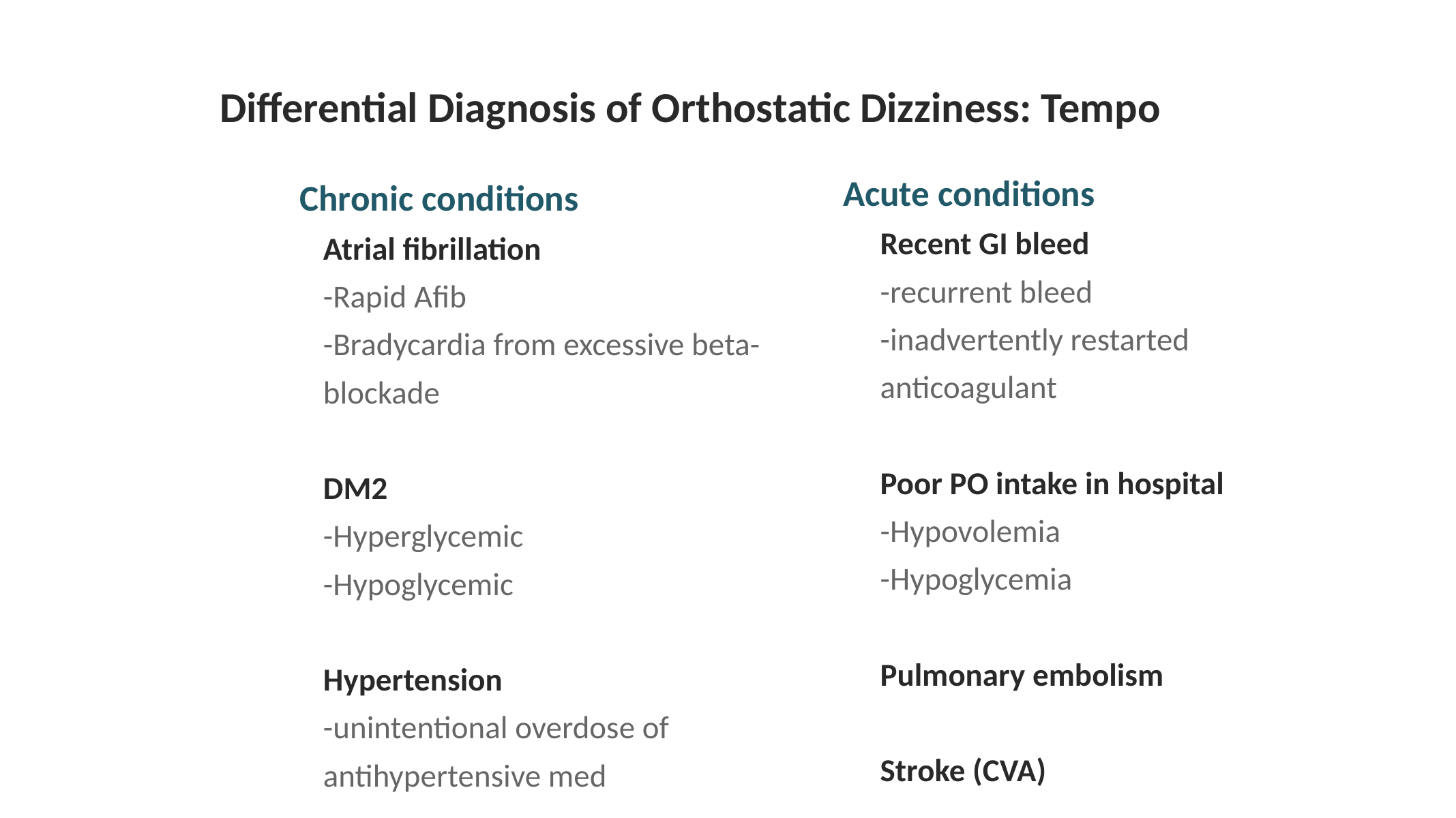

Differential Diagnosis of Orthostatic Dizziness: Tempo
Acute conditions
Chronic conditions
Recent GI bleed
-recurrent bleed
-inadvertently restarted anticoagulant
Poor PO intake in hospital
-Hypovolemia
-Hypoglycemia
Pulmonary embolism
Stroke (CVA)
Atrial fibrillation
-Rapid Afib
-Bradycardia from excessive beta-blockade
DM2
-Hyperglycemic
-Hypoglycemic
Hypertension
-unintentional overdose of antihypertensive med

## Slide 7
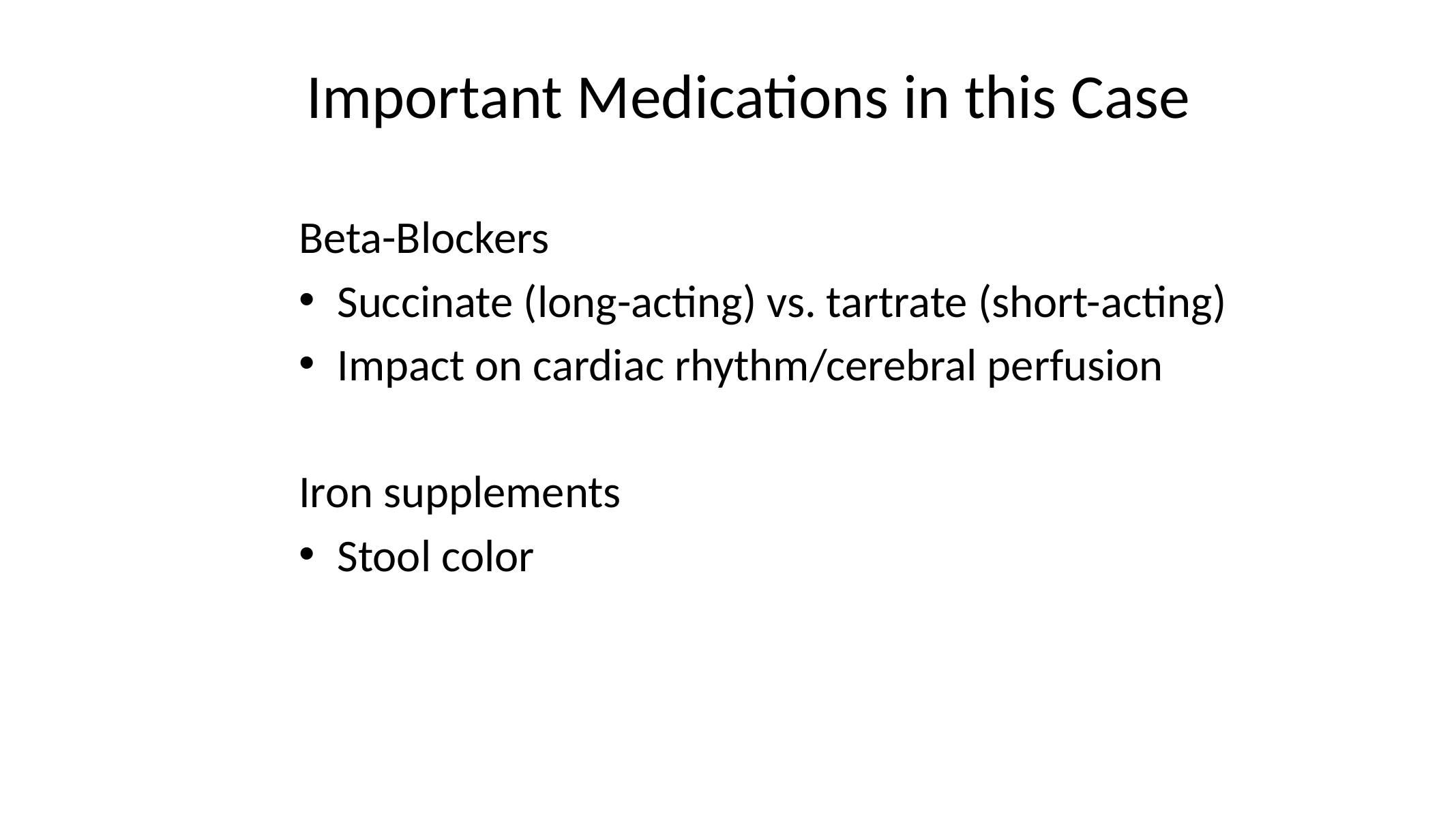

# Important Medications in this Case
Beta-Blockers
Succinate (long-acting) vs. tartrate (short-acting)
Impact on cardiac rhythm/cerebral perfusion
Iron supplements
Stool color

## Slide 8
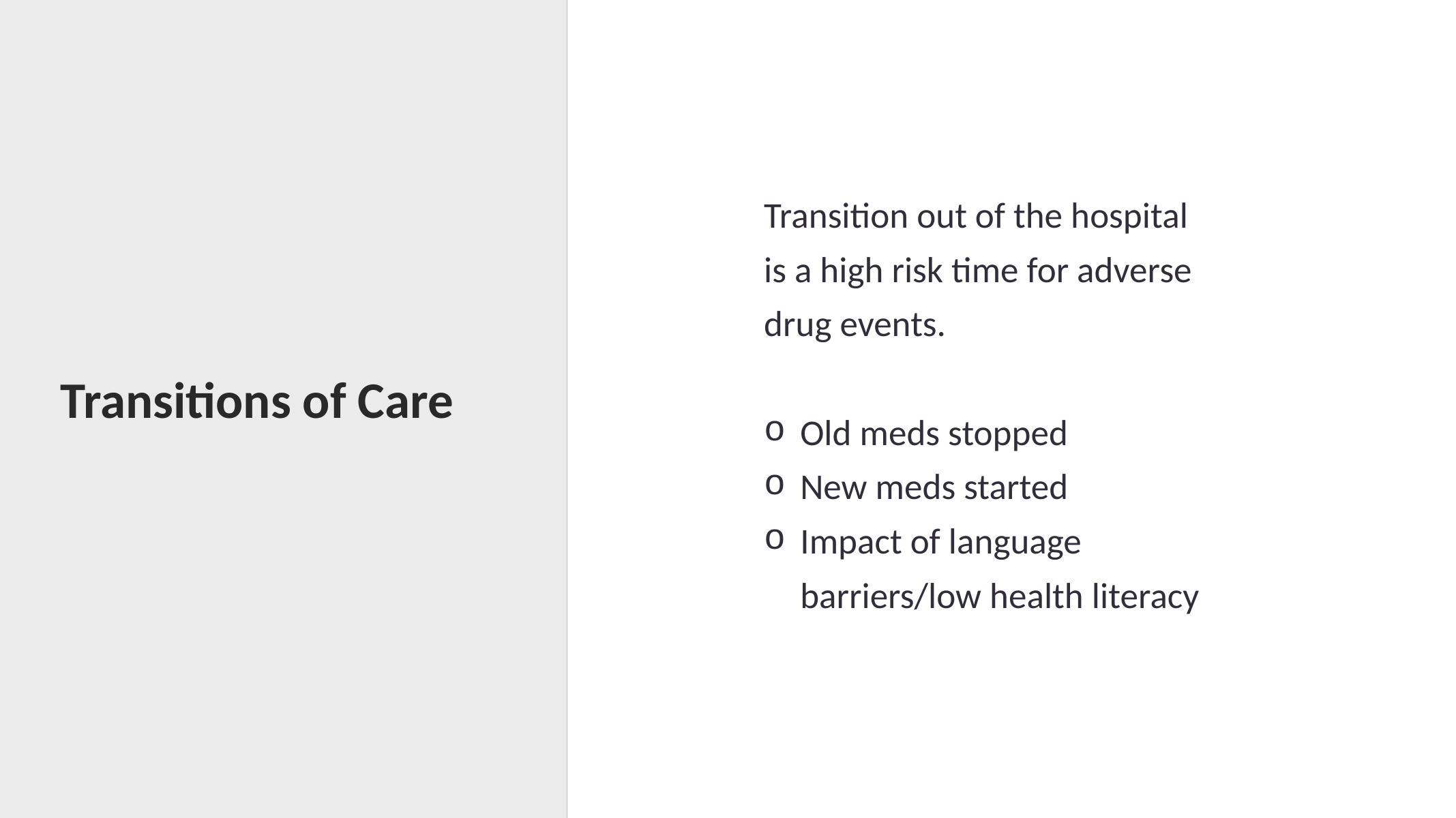

Transition out of the hospital is a high risk time for adverse drug events.
Old meds stopped
New meds started
Impact of language barriers/low health literacy
Transitions of Care

## Slide 9
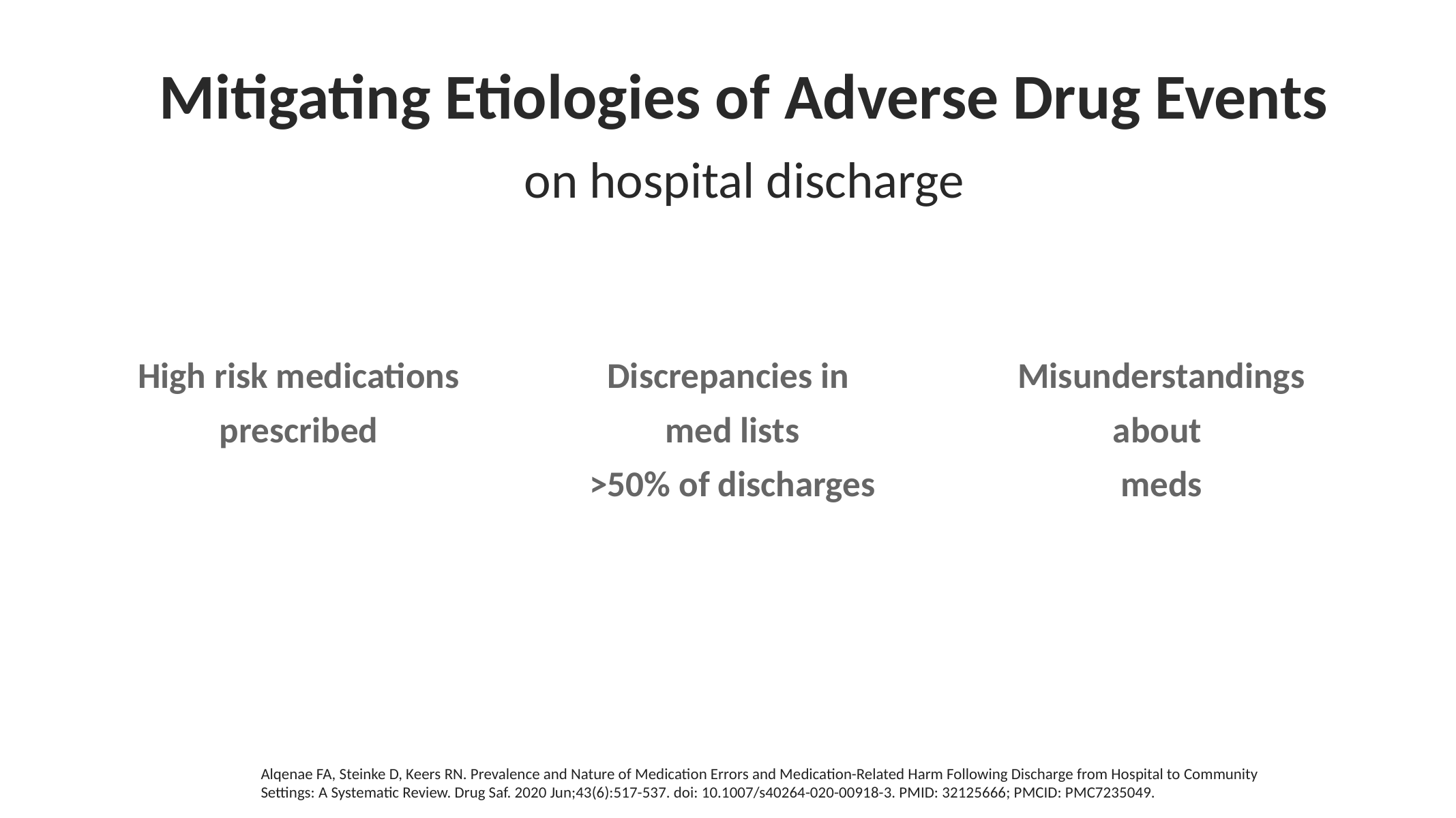

Mitigating Etiologies of Adverse Drug Events
on hospital discharge
High risk medications prescribed
Discrepancies in
med lists
>50% of discharges
Misunderstandings about
meds
Alqenae FA, Steinke D, Keers RN. Prevalence and Nature of Medication Errors and Medication-Related Harm Following Discharge from Hospital to Community Settings: A Systematic Review. Drug Saf. 2020 Jun;43(6):517-537. doi: 10.1007/s40264-020-00918-3. PMID: 32125666; PMCID: PMC7235049.

## Slide 10
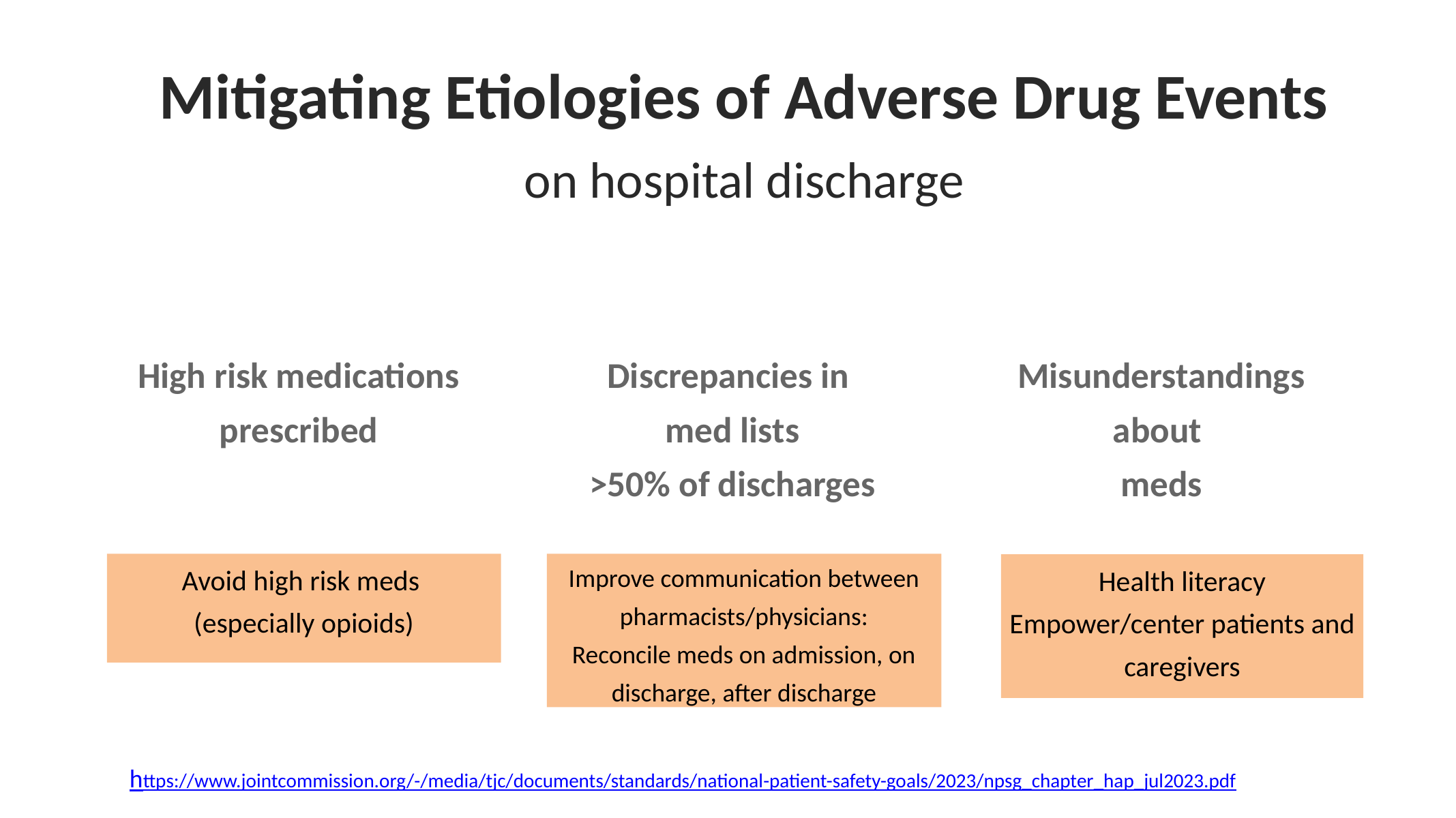

Mitigating Etiologies of Adverse Drug Events
on hospital discharge
High risk medications prescribed
Discrepancies in
med lists
>50% of discharges
Misunderstandings about
meds
Avoid high risk meds
(especially opioids)
Improve communication between pharmacists/physicians:
Reconcile meds on admission, on discharge, after discharge
Health literacy
Empower/center patients and caregivers
https://www.jointcommission.org/-/media/tjc/documents/standards/national-patient-safety-goals/2023/npsg_chapter_hap_jul2023.pdf

## Slide 11
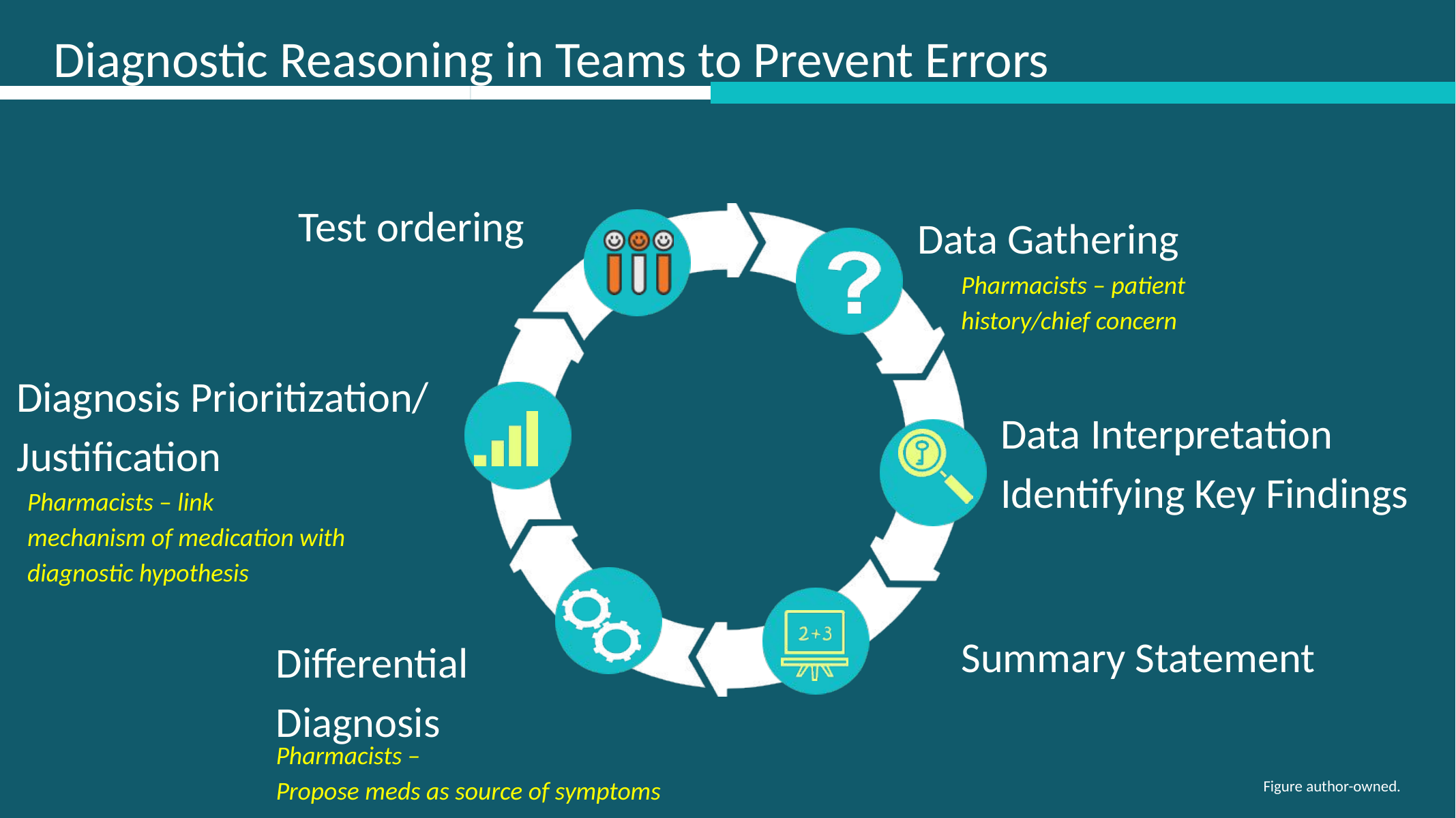

Diagnostic Reasoning in Teams to Prevent Errors
Test ordering
Data Gathering
Pharmacists – patient history/chief concern
Diagnosis Prioritization/
Justification
Data Interpretation
Identifying Key Findings
Pharmacists – link
mechanism of medication with
diagnostic hypothesis
Summary Statement
Differential Diagnosis
Pharmacists –
Propose meds as source of symptoms
Figure author-owned.

## Slide 12
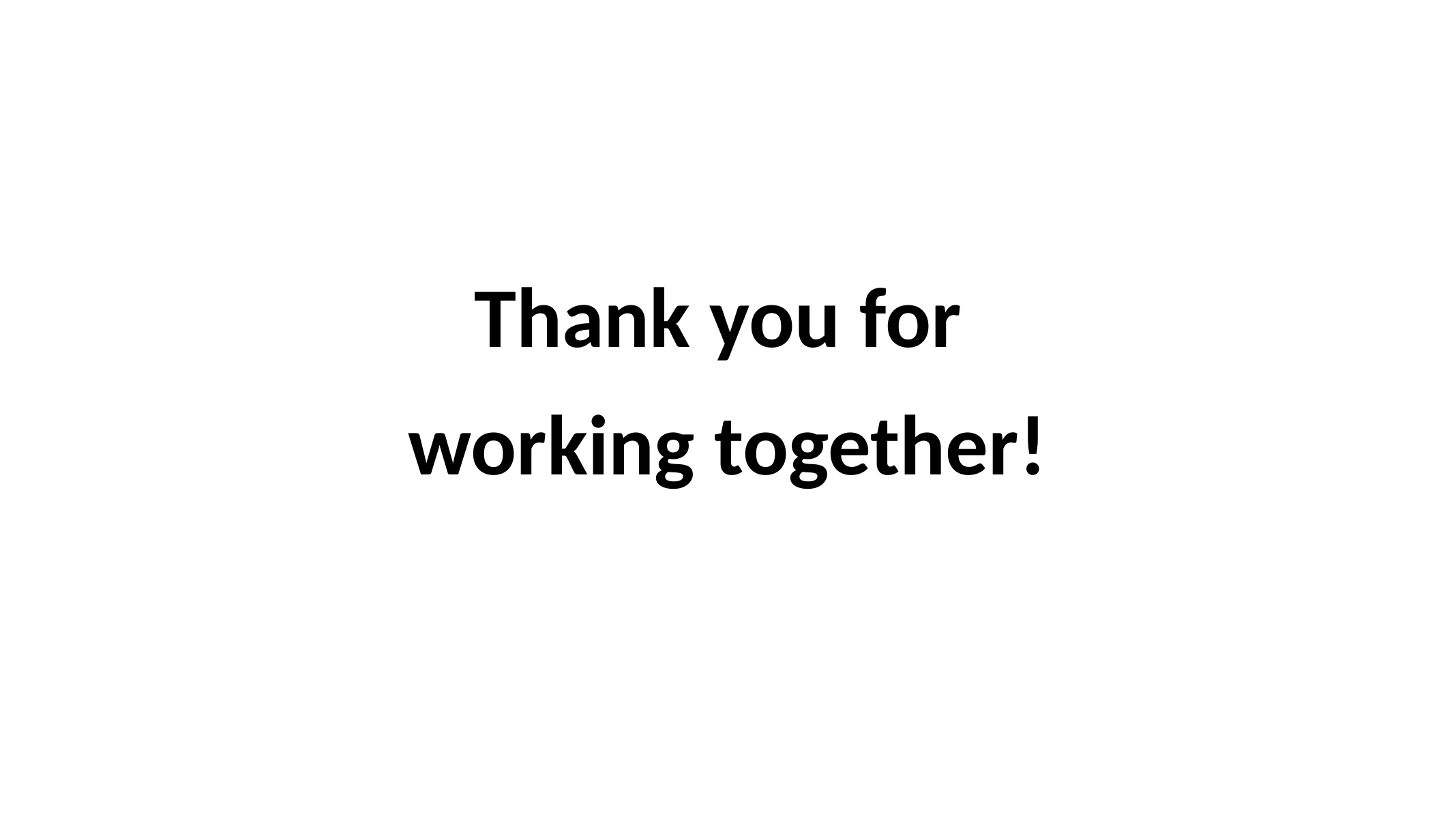

Thank you for
working together!
